# An International Consensus on the Design of Prospective Clinical–Translational Trials in Spatially Fractionated Radiation Therapy

**DOI:** 10.1016/j.adro.2021.100866

**Published:** 2021-12-11

**Authors:** Nina A. Mayr, James W. Snider, William F. Regine, Majid Mohiuddin, Daniel S. Hippe, José Peñagarícano, Mohammed Mohiuddin, Mahesh R. Kudrimoti, Hualin Zhang, Charles L. Limoli, Quynh-Thu Le, Charles B. Simone

**Affiliations:** a Department of Radiation Oncology, University of Washington School of Medicine, Seattle, Washington; b Tumor Heterogeneity Imaging and Radiomics Laboratory, University of Washington School of Medicine, Seattle, Washington; c Department of Radiation Oncology, University of Alabama at Birmingham School of Medicine, Birmingham, Alabama; d Department of Radiation Oncology, University of Maryland School of Medicine, Baltimore, Maryland; e Radiation Oncology Consultants and Northwestern Proton Center, Warrenville, Illinois; f Clinical Research Division, Fred Hutchinson Cancer Research Center, Seattle, Washington; g Moffitt Cancer Center, Tampa, Florida; h Radiation Oncology, Scottsdale, Arizona; i Department of Radiation Medicine, University of Kentucky College of Medicine, Lexington, Kentucky; j Department of Radiation Oncology, Northwestern University Feinberg School of Medicine, Chicago, Illinois; k Department of Radiation Oncology, University of California School of Medicine, Irvine, Irvine, California; l Department of Radiation Oncology, Stanford University, Stanford, California; m Department of Radiation Oncology, New York Proton Center, New York, New York

## Abstract

**Purpose:**

Spatially fractionated radiation therapy (SFRT), which delivers highly nonuniform dose distributions instead of conventionally practiced homogeneous tumor dose, has shown high rates of clinical response with minimal toxicities in large-volume primary or metastatic malignancies. However, prospective multi-institutional clinical trials in SFRT are lacking, and SFRT techniques and dose parameters remain variable. Agreement on dose prescription, technical administration, and clinical and translational design parameters for SFRT trials is essential to enable broad participation and successful accrual to rigorously test the SFRT approach. We aimed to develop a consensus for the design of multi-institutional clinical trials in SFRT, tailored to specific primary tumor sites, to help facilitate development and enhance the feasibility of such trials.

**Methods and Materials:**

Primary tumor sites with sufficient pilot experience in SFRT were identified, and fundamental trial design questions were determined. For each tumor site, a comprehensive consensus effort was established through disease-specific expert panels. Clinical trial design criteria included eligibility, SFRT technology and technique, dose and fractionation, target- and normal-tissue dose parameters, systemic therapies, clinical trial endpoints, and translational science considerations. Iterative appropriateness rank voting, expert panel consensus reviews and discussions, and public comment posting were used for consensus development.

**Results:**

Clinical trial criteria were developed for head and neck cancer and soft-tissue sarcoma. Final consensus among the 22 trial design categories each (a total of 163 criteria) was high to moderate overall. Uniform patient cohorts of advanced bulky disease, standardization of SFRT technologies and dosimetry and physics parameters, and collection of translational correlates were considered essential to trial design. Final guideline recommendations and the degree of agreement are presented and discussed.

**Conclusions:**

This consensus provides design guidelines for the development of prospective multi-institutional clinical trials testing SFRT in advanced head and neck cancer and soft-tissue sarcoma through in-advance harmonization of the fundamental clinical trial design among SFRT experts, potential investigators, and the SFRT community.

## Introduction

Spatially fractionated radiation therapy (SFRT) is a complex concept of increasing clinical, experimental, and translational interest. Pilot studies have shown low rates of toxicities and unexpectedly high tumor responses to SFRT in bulky metastatic and primary tumors.^1^, ^2^, ^3^, ^4^, ^5^, ^6^, ^7^, ^8^, ^9^, ^10^, ^11^, ^12^, ^13^ The observed enhanced tumoricidal effects of SFRT are thought to be related to the vastly inhomogeneous dose distributions.^4^^,^^14^^,^^15^ The inherent advantages of ablative stereotactic radiosurgery or stereotactic radiation therapy like dose “peaks,” dispersed throughout the tumor, combined with interlaced low dose in the “valleys,” suited to preserve the tumor microenvironment and vasculature, are postulated to promote bystander and abscopal effects as potential underlying mechanisms for higher tumor response.^15^, ^16^, ^17^, ^18^, ^19^, ^20^ Such mechanisms are of particular interest in the current era of immune-modulating agents that are increasingly combined with radiation therapy.^21^

Multiple single-institution studies have shown high response and local control rates in cohorts treated largely palliatively with GRID and Lattice SFRT.^1^, ^2^, ^3^, ^4^ These studies established the initial dose-response relationships and the need for combining SFRT with fractionated conventional radiation therapy.^2^^,^^3^ More recently, smaller, disease-specific pilot studies in head and neck (H&N),^5^^,^^6^^,^^13^ lung,^7^ and cervical^8^ cancer, sarcoma,^9^^,^^10^^,^^12^ and melanoma^11^ have advanced the SFRT concept from palliative treatment to curative-intent therapy of bulky primary tumors and showed similarly promising local control. They have also broadened the SFRT experience from reporting on palliative responses to providing early data on favorable survival outcomes in patients with nonmetastatic cancer^5^, ^6^, ^7^, ^8^ while affording longer follow-up for adverse effect assessment, which corroborates SFRT's overall favorable toxicity profile.

Thus, SFRT has the potential to broaden radiation therapy options for patients with locally advanced bulky primary, recurrent, and/or metastatic malignancies, for which with current techniques, the deliverable tumor dose is often severely compromised by normal-tissue tolerance limits.

Based on these hypothesis-generating studies,^5^, ^6^, ^7^, ^8^, ^9^, ^10^, ^11^, ^12^, ^13^ well-designed prospective clinical trials, preferably multi-institutional studies, are now needed to rigorously study SFRT as a modality. There is also an unmet need to evaluate feasibility of SFRT across institutions and technology platforms and to further elucidate its underlying biologic mechanisms through correlative translational science within the trial design. To our knowledge, no such phase 3 or multi-institutional prospective trials have been conducted in SFRT to date.

Design of such trials is challenged by SFRT's profound departure from familiar uniform-dose concepts and the requirement of complex biological modeling and nonintuitive dose-prescription metrics. Furthermore, SFRT platforms, techniques, dose and fractionation schemes have been variable. Currently, 2 profoundly different SFRT technologies are in use. GRID therapy,^2^^,^^4^^,^^22^ the first SFRT technology, developed using GRID collimators, has since evolved into an multileaf collimator (MLC)-based platform^4^^,^^6^^,^^13^ with a different dose profile. Most recently, Lattice therapy, a 3-dimensional form of SFRT,^23^, ^24^, ^25^ has emerged. These variabilities likely introduce additional inconsistencies that can hamper trial design and interpretation and therefore require thorough assessment, consensus, and standardization for the specific disease site under investigation. The challenge is compounded by a paucity of reviews^14^^,^^26^ and an absence of meta-analyses to provide guidance to investigators for trial design.

To address these obstacles toward development of multi-institutional prospective trials in SFRT, we sought to establish an in-advance common understanding and consensus among SFRT experts regarding the major design parameters and feasibility requirements for SFRT trial development, informed by the collective disease-specific clinical experience and by physics and biology expertise. The consensus effort comprised 2 major candidate primary disease sites—H&N cancer and soft-tissue sarcoma (STS)—and focused on the full range of clinical trial design criteria, including eligibility, stratification, endpoints, prescription dose and fractionation, target- and normal-tissue dose parameters, SFRT technology and technique, systemic therapies, patient assessments, and correlative science investigations.

## Methods and Materials

This consensus effort was conducted as part of the activities of the Radiosurgery Society (RSS) GRID, Lattice, Microbeam and FLASH Radiation Therapy Working Groups that were established, subsequent to an inaugural 2018 RSS–National Cancer Institute Workshop,^14^ to advance understanding of the biology, physics, and technology, and the clinical applications of these emerging technologies.

A comprehensive literature search for GRID and Lattice SFRT was performed (Table 1). The literature was systematically reviewed for studies that reported clinical outcomes. Studies were critically appraised for entry criteria, treatment parameters, and outcome reporting; and were tabulated in literature evidence tables (Appendices E1 and E2).

**Table 1:** Synopsis of the consensus development process

Sequence	Process description		
1. Initial literature review	Search terms: Spatially fractionated radiation therapy, GRID therapy, Lattice therapy, dose fractionation, radiation, neoplasms/radiation therapy, neoplasms/pathology, tumor control Databases: PubMed, Web of Science, Cochrane Repeat literature search: April 2021		
	Tabulation of literature into evidence tables (Appendices 1-2)		
2. Development of initial clinical trial design criteria	Design criteria: Eligibility/exclusion, pretherapy, on-therapy, and posttherapy patient evaluations (for outcome endpoints), endpoints, stratifications, dose and technical radiation therapy factors, clinical feasibility of correlative of studies, concurrent therapies, and knowledge gaps that may be addressed in a trial		
	Performed by expert group of 3 leaders in general SFRT		
3. Voting round 1	Anonymous electronic rating of the appropriateness of the proposed trial design criteria: 21 categories of trial design questions with 1-11 subcriteria, (total of 75 for H&N cancer and 88 for STS): Voting scale 1-9* 1 knowledge-gap question 1 demographic expertise question		
	Voters: radiation oncologists, physicists, and biologists with clinical experience in SFRT in the disease site and/or or publications and/or scientific presentations		
4. Vote analysis and statistical model	Prioritization of agreement on the broader appropriateness categories (*appropriate, may be appropriate*, or *not appropriate*)* while maintaining the nuancing of the 1-9 scale		
	Agreement categories: *high, moderate*, and *low*†		
5. Review/discussion of voting results by disease-specific consensus expert panel (“panel”)	Panel members: 3 radiation oncologists, 1 physicist, and 1 biologist with SFRT publications or scientific presentations in the specific disease site, physics, or biology, respectively		
	Consensus development based on voting statistics, literature and the panel's clinical/scientific experience		
	Formal consensus video conference call(s) and consensus communications (email, phone)		
6. Iterative voting round(s)	Implemented for trial criteria with persistently low agreement, or new trial criteria identified by the panel		
7. Rereview/discussion of voting results	As in step 5 (with or without video conference call)		
8. Draft guideline development	Guideline draft and review by panel		
9. Public comments	Public comment posting for 2 weeks per disease site (by RSS)		
10. Repeat literature review	As in step 1		
11. Review/discussion of public comments	Review of anonymized public comments, as in steps 5 and 7; guideline revisions as indicated		
12. Final guideline	Development of final guideline by panel		

	Voting scale		
Voting rank	1 2 3	4 5 6	7 8 9
Voting category	Not appropriate for clinical trial design	May be appropriate for clinical trial design	Appropriate for clinical trial design

Vote agreement	Definitions†		
High	Percentage agreement ≥67% AND if there is any disagreement, it is by at most 1 voting category		
Moderate	60%-67% agreement OR agreement ≥67% but votes in both *appropriate* and *not appropriate* vote categories		
Low	Percentage agreement <60%		

*Abbreviations:* H&N = head and neck; RSS = Radiosurgery Society; SFRT = spatially fractionated radiation therapy; STS = soft-tissue sarcoma.

⁎ Voting scale and categories: Within each voting category, 3 subranks (eg, 7, 8, and 9) signify ranking as lower, intermediate, and higher appropriateness, respectively.

† Details of vote agreement categories: Agreement on the rating of each clinical trial criterion was categorized as either *low, moderate,* or *high. Low agreement* was defined as the percentage of agreement on the broader appropriateness category (*appropriate, may be appropriate,* and *not appropriate*) of less than 60%. High agreement was defined as percent agreement > 67% on the appropriateness category AND no disagreement (if any was present) by more than 1 category. Thus, ratings of *appropriate* and *may be appropriate,* or *may be appropriate* and *not appropriate* for the same clinical trial criterion were allowable under *high agreement* if at least two-thirds agreed on a single appropriateness category, whereas ratings of both *appropriate* and *not appropriate* could not qualify for *high agreement,* regardless of the overall percentage of agreement. All others were classified as *moderate agreement.*

The overall process and rationale of the consensus procedure are described in detail in Table 1. Initial draft criteria for clinical trial design were developed among a group of leading SFRT experts. Design criteria were based on pertinent clinical trial principles according to the categories outlined in Table 2 and then were tailored to the individual primary tumors.

**Table 2:** Clinical trial design categories

Design categories	Subcategories
Eligible disease sites	Primary tumor sites
Eligibility/exclusion criteria	Disease stage, tumor size/extent/invasion
	Histology, molecular markers
	Prior treatment
	Patient factors: age, performance status
Stratifications	
Pretreatment evaluations	Clinical
	Imaging
	Histologic investigations
Radiation therapy: SFRT	SFRT dose
	SFRT target volume
	SFRT OAR constraints
	SFRT technique
Radiation therapy: Conventional external beam radiation therapy	cERT dose and fractionation
	cERT technique
	cERT OAR constraints
On-therapy evaluations	Clinical
	Laboratory
	Imaging
	Patient-reported outcomes
	Translational studies (evaluation of clinical feasibility)
Systemic therapy	Cytotoxic agents and timing
	Immunotherapy
Posttherapy evaluations	Clinical
	Imaging
	Patient-reported outcomes
Knowledge gaps	Clinical
	Physics
	Biology/translation science

*Abbreviations:* cERT = Conventional external beam radiation therapy; OAR = organ at risk; SFRT = spatially fractionated radiation therapy.

For each disease site, an anonymous 22-question voting survey with 1 to 11 subcriteria questions and the respective literature evidence tables (Appendices E1 and E2) were distributed to national and international experts with publications, scientific presentations, and/or clinical SFRT practice in the respective disease site (voting round 1). Voting for appropriateness and prioritization of each trial-design criterion was performed on a 1 to 9 scale (Table 1) with additional optional free-text comments.

For the analysis of voting results, in addition to descriptive statistics quantifying the appropriateness of each design criterion, a statistical model was developed (Table 1) to quantitate the level of agreement among an overall low number of voters to address the challenge of the relatively small number of existing disease-specific SFRT experts.

A disease-specific consensus expert panel of 3 radiation oncologists, a physicist, and a biologist with scientific SFRT publications and/or presentations was established for each disease site to develop the consensus recommendations and guideline. The aggregated voting round 1 results were circulated among each disease-specific panel, reviewed, and discussed in sequential conference calls and communications, using modified Delphi technique^27^ principles (Table 1). Remaining controversies and/or new trial design considerations were subjected to a second voting round in H&N cancer, followed by iterative panel review and discussions. For STS, only 1 voting round was required. Detailed voting results and panel discussions are presented in the respective consensus tables (Appendices E3 and E4).

The drafts of the resulting consensus guidelines were posted on the RSS website for public comment. After review of the comments, the panel finalized the guideline as summarized in this article. The detailed guidelines are presented in Appendices E5 and E6.

## Consensus Guideline Recommendations and Discussion

### SFRT Clinical Trial Design Consensus Guideline for H&N Cancer

The clinical trial design recommendations for H&N cancer were guided by 3 SFRT outcome studies of multiple disease sites containing H&N cancer patients^2^, ^3^, ^4^ and 3 disease-specific series of only H&N cancer^5^^,^^6^^,^^13^ (Appendix E1). These studies showed high local and regional control rates in the neck of 79% to 92% and survival rates of 50% to 79% in patients with far-advanced tumors^5^^,^^6^ that compared favorably with the regional control rates of 25% to 66% and survival of 30% reported with conventional radiation therapy or radiation therapy and chemotherapy.^28^, ^29^, ^30^, ^31^, ^32^ These observations provided rationale to test SFRT rigorously in multi-institutional trials of bulky neck disease.

### Eligibility

The consensus on eligibility and exclusion criteria is summarized in Table 3. Recommendations for eligibility aimed to establish, based on patient characteristics in the pilot studies^5^^,^^6^ (Appendix E1), a uniform patient cohort of oropharynx, larynx (high consensus), and nasopharynx (moderate consensus) primaries with bulky lymph node involvement. Eligibility should be guided by the lymph node status, not the status (T-stage) of the primary (high consensus), emphasizing that the majority of reported clinical experience with SFRT in H&N cancer is in the treatment of bulky lymph nodes.^2^, ^3^, ^4^, ^5^, ^6^^,^^13^ Patients with any T-stage oropharynx, larynx, or nasopharynx cancer and N3 nodal stage are eligible (Table 3). Similarly, stage N3 skin primaries may be included (high consensus). Eligible histology includes squamous cell carcinoma, based on the majority of published experience,^5^^,^^6^^,^^13^ both human papillomavirus (HPV) (P16) positive and negative (moderate consensus). Uncommon primaries and uncommon or highly radiosensitive histologies are excluded to minimize confounding variables that may hamper interpretation of outcome results (high consensus).

**Table 3:** Eligibility, exclusions, and stratifications in H&N cancer SFRT trials

Eligibility criteria	
Disease sites	Oropharynx, hypopharynx, supraglottic larynx, glottic larynx, and nasopharynx primary tumors*
Stage, tumor size	Stage N3 tumors with any T-stage Single lymph node or matted nodes with lymph node size totaling >6 cm
Histology and tumor markers	Squamous cell carcinoma, HPV negative or HPV positive
Prior therapy	No prior therapy†
Patient factors	Age >18 y No upper age limit if eligible based on performance status

Exclusion criteria	
Disease sites	Salivary gland tumors, paranasal sinus tumors*
Histology and tumor markers	Tumors considered radiosensitive, such as lymphoma, multiple myeloma, and leukemic infiltrates
Tumor stage/extent	Both carotid artery invasion and skin involvement Both carotid artery invasion and prior radiation
Prior therapy	Recurrent tumors after prior radiation therapy Recurrent tumors after prior surgery† Prior chemotherapy for H&N cancer
Patient factors	Active scleroderma (systemic sclerosis)

Stratifications	
T-stage grouping	Stage T1/2 vs T3/4
HPV status	HPV negative vs positive
SFRT technology	GRID vs Lattice‡

*Abbreviations:* H&N = head and neck; HPV = human papillomavirus; SFRT = spatially fractionated radiation therapy.

⁎ Primary skin cancer with stage N3 lymph node involvement is eligible. There is currently insufficient clinical evidence in favor of specific individual H&N primary sites for inclusion into SFRT trials (high consensus). Uncommon primary sites, such as salivary gland and paranasal sinus tumors, should be excluded because of their different spread pattern, often variable histology, and overall low incidence (high consensus).

† Recurrent tumors after prior surgery may be eligible if recurrence consists of bulky neck nodes that were not previously irradiated.

‡ If Lattice therapy is used in subsequent trials, stratification may include GRID versus Lattice therapy technologies.

The panel unanimously recommended exclusion of tumors with both carotid invasion and skin involvement, or both carotid invasion and prior radiation therapy, based on fatal carotid bleeding after SFRT in a patient with carotid invasion and prior radiation,^6^ and in a patient with carotid invasion and skin involvement (unpublished).

Patients with prior radiation therapy (moderate consensus), prior surgery, and/or (induction) chemotherapy (high consensus) are excluded, with the exception of prior surgery with no prior radiation to the target region. A separate clinical trial (to follow an initial trial of patients without previous radiation therapy) is suggested for patients with postradiation recurrences.

### Stratifications

Stratifications according to T-stage and HPV status are recommended based on their strong influence on outcome. Stratification by SFRT technology—for example, GRID versus Lattice therapy (if Lattice is used in the future)—is recommended because of dosimetric differences (Table 3).

### Endpoints

Local control and treatment related toxicity are recommended as primary endpoints. The feasibility of delivering SFRT according to the dosimetric and physics specifications^22^ (see the section “SFRT: Dose”), disease-specific survival, overall survival, and quality of life (QOL) outcomes represent additional endpoints.

### Radiation therapy

#### SFRT: Dose

Based on the outcome data^2^, ^3^, ^4^, ^5^, ^6^^,^^13^ (Appendix E1), the preferred SFRT dose is 15 Gy/1 fraction to the bulky lymph node(s). In 2 of the 3 H&N cancer cohorts, 15 Gy/1 fraction was most commonly used and was associated with high local control and low toxicity,^5^^,^^13^ providing the basis for this recommendation. Although a schedule of 20 Gy/1 fraction was used in 1 cohort^6^ and in small proportions of patients in other studies,^4^^,^^5^^,^^13^ 20 Gy has been more commonly used in the palliative setting.^2^, ^3^, ^4^ Therefore, and in the absence of a dose response relationship favoring 20 Gy, the panel considered 15 Gy/1 fraction the preferred dosing regimen for an initial SFRT trial (high consensus). The dose at the GTV periphery, which is generally in the range of 3 Gy for a 15-Gy SFRT dose,^33^ should be reported.

Standardization of the GRID dose prescription, defined as the peak dose, is required. In addition, dosimetric and geometric characteristics of the heterogeneous dose distribution, such as dose volume histogram characteristics (D10, D50, D90) and peak-to-peak distance, should be reported according to guidelines further described in the recent GRID physics and dosimetry white paper.^22^ Owing to the different SFRT technologies (eg, collimator-based and MLC-based GRID) with different dose distributions,^34^ the equivalent uniform dose (EUD) for H&N squamous cell carcinoma (using α/β = 10 Gy) and for normal tissues (generally α/β = 3 Gy) must be determined for any trial regimen. Principles of EUD computation in SFRT, which favor the modified linear quadratic model, and tumor cell sensitivity considerations are described in the recent SFRT physics guideline publications.^22^^,^^25^

#### SFRT: Target volume

The SFRT target should consist of the involved nodal mass (GTV)^4^, ^5^, ^6^^,^^13^ by imaging-based delineation, without an additional margin (high consensus).

#### SFRT: Normal organ-at-risk structures

Based on published data^4^, ^5^, ^6^^,^^13^ and the panel's clinical experience, critical normal organ-at-risk (OAR) structures include the spinal cord, brain stem, and optic chiasm (high consensus). Consideration of the brachial plexus, carotid artery, and mandible as OARs may be appropriate (moderate consensus). The addition of planning organ at risk volume (PRV) margins to the OAR structures can be considered, particularly for the spinal cord and brain stem (moderate consensus).

#### SFRT: Technique

Based on current published data,^4^, ^5^, ^6^^,^^13^ GRID technologies are preferred. Collimator-based and MLC-based GRID therapy may be applied within the same trial under the condition that the EUD is comparable. While there was overall support for Lattice therapy as an SFRT technology in the future, to the panel's knowledge, there were no published outcome data on Lattice SFRT in H&N cancer at the time of this writing. Whereas such published experience is expected to emerge, at this time, the panel favored GRID therapy technologies for an initial clinical trial.

#### Conventional ERT: Dose and technique

Conventionally fractionated external beam radiation therapy (cERT) must be given after SFRT, because it has been demonstrated that tumor response is inferior when cERT is omitted.^2^^,^^3^ The cERT should start within 72 hours of the SFRT fraction.

For the cERT component of treatment, conventional definitive dose regimens, specific to the H&N disease site, are prescribed. PTV doses are generally in the range of 70 to 72 Gy (2-2.12 Gy/fraction) to the gross tumor, 60 to 63 Gy to the high-risk subclinical target, and 50 to 56 Gy to the low-risk subclinical target (high consensus). Reduction of the definitive cERT dose below standard dose levels is not recommended because a reduced response rate of only 25% was reported with cERT doses of less than 75% of the planned definitive dose.^13^ The use of intensity modulated radiation therapy is encouraged (high consensus). A simultaneously integrated boost is acceptable for bulky involvement; however, if used, the dose to the SFRT GTV should be limited to 69.6 Gy.

#### Conventional ERT: OAR constraints

Dose constraints to OARs for the cERT component follow those of standard practice without consideration of the dose contribution from the SFRT (high consensus). The SFRT contributions to OARs must be addressed during the planning of the SFRT component of treatment (see the section “SFRT: Normal organ-at-risk structures”).

### Systemic therapy

#### Agents and timing

Chemotherapy and targeted systemic therapy agents that are typically considered appropriate in conjunction with standard-fractionation radiation therapy for H&N cancer are acceptable for a clinical trial (high consensus). These agents typically include but may not be limited to platinum-based chemotherapies, taxanes, and cetuximab. Chemotherapy can be given concurrently with radiation therapy for the cERT component of treatment. However, systemic therapy should not be given during SFRT (high consensus). Typical schedules that have been used consist of delivering SFRT (without systemic therapy) on a Friday, followed by cERT and concurrent systemic therapy start within 72 hours on the following Monday.

#### Immunotherapy

In the absence of published experience with combinations of SFRT and immunotherapy in H&N cancer, there was no consensus among voters on this combination. The panel favored not to include immunotherapy for an initial trial (moderate consensus) and to test combined therapy in a subsequent trial using guidance from the ablative stereotactic radiation therapy and immunotherapy experience, and future SFRT and immunotherapy experience.

### Evaluations and assessments

Patient evaluations before, during, and after treatment are recommended according to the standard of care for H&N cancer. These are detailed in Table 4 along with additional trial-specific assessments, specifically QOL assessments, patient-reported outcomes, and imaging. Cone beam computed tomography imaging during treatment can be incorporated into trials to establish criteria for intratreatment response assessment and adaptive therapy that may be required in SFRT.^8^

**Table 4:** Pretherapy, on-therapy, and posttherapy assessments in H&N SFRT trials

Assessments	Evaluation/test	Pretherapy	On-therapy	Posttherapy
Clinical	H&N examination	**√**	√*	**√**†
	Fiberoptic laryngoscopy	**√**^‡^	**√**^‡^	**√**†^,‡^
	Toxicity assessment	n/a	√*	**√**†
Imaging	CT maxillo/facial/neck	**√**	n/a	**√**§
	MRI maxillo/facial/neck	**√**	n/a	**√**§
	CT chest (including liver)	**√**	n/a	**√**^‡^
	Swallowing study	**√**^‡^	n/a	**√**^‡^
	PET/CT	**√**	n/a	**√**§
	On-board imaging (CBCT)	n/a	**√**ǁ	n/a
Laboratory	CBC	**√**	**√**^‡^	**√**^‡^
	Blood chemistries	**√**	**√**^‡^	**√**^‡^
Histology	HPV	**√**	n/a	n/a
Correlative studies	Blood collection	**√**	**√**¶	**√**¶
	Urine collection	**√**	**√**¶	**√**¶
	Tumor biopsy	**√**#	—	—
	Functional/molecular imaging	**√**⁎⁎	**√**⁎⁎	**√**⁎⁎
Patient-reported outcomes	QOL assessment	**√**	**√**	**√**†

*Abbreviations:***√** = recommended; **√**^‡^ = recommended if clinically indicated; — = not recommended; CBC = complete blood count; CBCT = cone beam computed tomography; CT = computed tomography; H&N = head and neck; HPV = human papillomavirus; MRI = magnetic resonance imaging; n/a = not applicable; PET = positron emission tomography; QOL = quality of life; SFRT = spatially fractionated radiation therapy.

⁎ Weekly.

† Routine follow-up every 3 months in years 1 to 2, and every 4 to 6 months in years 3 to 5.

§ PET/CT, or alternatively (if PET/CT is unavailable), maxillo/facial/neck CT or MRI.

ǁ CBCT imaging during treatment can be included as response assessment.

¶ Feasible weekly or at prospective time points and dose levels during or after treatment.

# Tissue from pretherapy biopsies may be procured for correlative studies.

⁎⁎ Functional imaging can be added to a diagnostic imaging session pretherapy and posttherapy and as additional imaging prospectively scheduled at various time points and dose levels during radiation therapy.

The outcome endpoint of local/regional control in the neck is important but can be challenging to definitively characterize because of interinstitutional variability in response assessment and in the use and timing of postradiation neck dissection. Determination of local control should be based on the 3-month posttherapy positron emission tomography/computed tomography (PET/CT), using established response criteria; and on the need for postradiation neck dissection, including pathologic response at the time of neck dissection.

To enable translational correlative science studies, specimen collection of blood and urine multiple times during radiation therapy should be strongly considered (high consensus). Although pretherapy tumor biopsies are available for correlative studies, tumor tissue sampling during the treatment course was considered not clinically practical or feasible based on the potential clinical risk. If possible and available, advanced functional and molecular imaging techniques such as vascular and metabolic imaging may provide noninvasive and non-tissue-altering approaches to characterize changes in functional tissue properties in response to SFRT during and after treatment.^35^, ^36^, ^37^, ^38^ Posttherapy patient-reported and QOL outcomes are recommended (high consensus).

## SFRT Clinical Trial Design Consensus Guideline for STS

As for H&N cancer, trial design recommendations for STS were based on multidisease series that included sarcoma patients and disease-specific series (Appendix E2). This experience comprised 2 studies of largely palliatively treated cohorts that contained sarcoma patients.^2^, ^3^, ^4^ Disease-specific series of definitively treated patients with STS have been presented in abstract form,^9^^,^^10^ and 1 outcome study^12^ was recently published. These studies showed high response rates, local control rates of 85% to 100%,^9^, ^10^, ^11^, ^12^ and a limb-sparing rate of 93%^9^ in bulky (>8-10 cm) sarcomas, which overall exceeded the outcomes of standard therapy.^39^, ^40^, ^41^ In contrast, with conventional preoperative radiation therapy, few patients with bulky sarcomas attain local control,^42^ and overall outcomes are poor. In high-grade sarcomas, median treatment-induced necrosis is only 50%,^43^ well below the recognized tumor control and survival predictor of ≥90% necrosis.^39^, ^40^, ^41^, ^42^, ^43^, ^44^ Collectively, the favorable SFRT pilot results^9^^,^^10^^,^^12^ and the challenge in improving outcomes with other strategies, including more toxic dose escalation^42^ or intensified chemotherapy,^45^ provide justification for the development of multi-institutional SFRT trials in STS.

### Eligibility

Eligibility and exclusion criteria are summarized in Table 5. Eligibility recommendations aim to establish a uniform patient cohort of bulky extremity sarcomas, the most common presentation, which also have the most SFRT pilot experience.^2^^,^^3^^,^^12^ This eligibility profile reflects that of the major prior randomized sarcoma trials using conventional radiation therapy.^40^^,^^41^^,^^46^ The panel considered it important to maintain a patient population that is consistent with these trial cohorts to allow comparison of SFRT outcomes with those of conventional radiation therapy.

**Table 5:** Eligibility, exclusions, and stratifications in sarcoma SFRT trials

Eligibility criteria	
Disease sites	Patients with primary sarcomas of extremities, to be treated with preoperative radiation therapy*
Stage, tumor size	Unresectable, stage IB-IIIB, bulky tumors ≥8 cm in largest diameter†
Histology and tumor markers	Undifferentiated pleomorphic sarcoma, myxoid liposarcoma, or leiomyosarcoma (high consensus)‡ Grade 2-3
Prior therapy	None except neoadjuvant chemotherapy
Patient factors	Age >18 y; upper age limit of 85 y may be appropriate (moderate consensus)

Exclusion criteria	
Disease sites	Less common primary sites, such as head and neck, intra-abdominal, or retroperitoneal sites
Histology and tumor markers	Rhabdomyosarcoma; Ewing sarcoma; chondrosarcoma, Kaposi sarcoma, and angiosarcoma; malignant peripheral nerve sheath tumor§ Grade 1
Tumor stage/extent	Tumors <8 cm in largest diameter
Prior therapy	Recurrent tumors after prior radiation Recurrent tumors after prior surgery Recurrent tumors after prior chemotherapy
Patient factors	Scleroderma (systemic sclerosis)║

Stratifications	
Tumor bulk	Largest dimension ≤12cm vs >12 cm
Neoadjuvant chemotherapy	Neoadjuvant chemotherapy vs none

*Abbreviation:* SFRT = spatially fractionated radiation therapy.

⁎ Less common primary sites, such as head and neck, intraabdominal, or retroperitoneal sites, should be excluded to reduce unnecessary variability (high consensus).

† Inclusion of patients with lymph node involvement (which is rare) may be appropriate (high consensus).

‡ This eligibility profile reflects that of major randomized prior trials in sarcoma with conventional radiation.

§ Although some of these histologies have been treated with SFRT, their different natural disease course and rarity was deemed to add confounding variability to an SFRT clinical trial cohort.

║ Exclusion because of high risk of toxicities, particularly in skin and subcutaneous tissues (high consensus).

Patients with stage IB-IIIB, grade 2 to 3, bulky ≥8 cm STS, who are planned to be treated with preoperative radiation therapy (high consensus), are eligible. Both neoadjuvant chemotherapy and no chemotherapy are permitted, reflecting the current practice pattern in STS. Prior resection, prior radiation therapy, and scleroderma (associated with higher risk of toxicities, particularly in subcutaneous and skin regions) are exclusion criteria (high consensus).

### Stratifications

Tumor bulk, using the largest imaging-based tumor diameter of <12 cm versus ≥12 cm, and use of neoadjuvant chemotherapy versus no chemotherapy (see the section “Concurrent systemic therapy: Agents and timing”), should be stratified (Table 5). Owing to the redundancy in molecular pathways, molecular marker-based subclassification or stratification is not recommended for an initial trial in this rare disease.

### Endpoints

The feasibility of delivering SFRT according to the dosimetric and physics specifications^22^ (see the section “SFRT: Dose”), primary tumor response (by imaging and pathologic response), and resectability are suitable primary endpoints. Local recurrence-free, metastasis-free, overall survival, and QOL outcomes present additional endpoints.

### Radiation therapy

#### SFRT: Dose

A dose range of 15 to 18 Gy in 1 fraction is an appropriate dosing regimen for clinical trials (high consensus), with the higher dose favored. The EUD of the SFRT regimen must be determined for sarcoma and for the normal tissues as described for H&N cancer.

#### SFRT: Target volume

The SFRT target volume, based on clinical experience,^9^^,^^10^^,^^12^ is the GTV of the primary tumor without an additional margin.

#### SFRT: OAR constraints

Consideration should be given to excluding sensitive neural structures such as the brachial plexus from the SFRT volume and beam path (high consensus). Exclusion of OARs with a 1-cm margin, usually through secondary collimation with MLC blocking, are expected to achieve a negligible SFRT dose. It is also recognized that this may not be possible if these OARs are involved with a tumor. The skin surface dose from SFRT should be limited to <150% of the prescribed SFRT dose based on the STS brachytherapy experience.^47^

#### SFRT: Technique

For an initial clinical trial, GRID therapy is the technology of choice, because all currently published clinical experience is in GRID therapy^2^^,^^3^^,^^9^^,^^10^^,^^12^ (high consensus). Although published data with Lattice in STS are currently lacking, Lattice therapy may be appropriate in subsequent trials.

#### Conventional ERT: Dose and technique

The cERT dose, following the SFRT fraction, is 50 to 50.4 Gy / 25 to 28 fractions to the PTV (high consensus) per RTOG trial regimens,^41^^,^^46^ using intensity modulated radiation therapy or a 3-dimensional conformal technique.^41^^,^^46^ As in standard-of-care radiation therapy, treatment to the entire extremity circumference must be avoided (high consensus).

Most commonly, the cERT course begins 1 to 2 days after SFRT^4^ and should start ideally within 3 days of the SFRT fraction. The interval between SFRT and cERT should be documented to elucidate any potential effects on outcome.

#### Conventional ERT: OAR constraints

Conventional dose constraints to critical normal tissues are applied. The dose contribution from the SFRT is not counted toward the dose constraints (moderate consensus). For concerns regarding normal-tissue doses, the OAR dose should be reduced up front when planning the SFRT by adjusting field size and shape (see the section “SFRT: OAR constraints”).

### Systemic therapy

#### Agents and timing

Neoadjuvant chemotherapy (prior to radiation therapy) and adjuvant chemotherapy after radiation therapy completion are acceptable. Agents considered acceptable in standard-of-care practice are allowable in clinical trials (high consensus).

Concurrent chemotherapy, delivered during the radiation therapy course, is not considered permissible in an initial clinical trial (high consensus). Concurrent chemotherapy is inconsistently and not widely used in current practice, providing a rationale for its omission, along with the potential to introduce confounding variables for the interpretation of tumor control and toxicity outcomes.

#### Immunotherapy

Immunotherapy was not considered to be recommended in an initial clinical trial (high consensus) but can be studied in subsequent trials or as a lead-in study as more combined STS radiation therapy and immunotherapy data emerge.

### Evaluations and assessments

Evaluations are presented in Table 6. Pretherapy standard workup includes magnetic resonance imaging or CT of the extremity and chest, abdomen, and pelvis CT or PET/CT for metastatic workup.

**Table 6:** Pretherapy, on-therapy, and posttherapy assessments in sarcoma SFRT trials

Assessments	Evaluation/test	Pretherapy	On-therapy	Postradiation therapy/preoperative	Surgical/ histologic	After completion of all therapy
Clinical	Clinical examination	**√**	√*	**√**†	n/a	**√**‡
	Toxicity assessment	n/a	√*	**√**†	n/a	**√**‡
Imaging	MRI (extremity)	**√**	n/a	**√**§	n/a	**√**§
	CT (extremity)	**√**	n/a	**√**§	n/a	**√**§
	CT Chest/abdomen/pelvis CT	**√**	n/a	n/a	n/a	**√ǁ**
	PET/CT	**√**	n/a	n/a	n/a	**√ǁ**
Laboratory	CBC	**√**	n/a	**√**	n/a	**√**
	Blood chemistries	**√**	n/a	**√**	n/a	**√**
Histology	Tumor necrosis	n/a	n/a	n/a	**√**¶	n/a
Correlative studies	Blood collection	**√**	**√**#	**√**	n/a	**√**
	Urine collection	**√**	**√**#	**√**	n/a	**√**
	Tumor biopsy/specimen	**√**⁎⁎	—	—	**√**⁎⁎	n/a
Patient-reported outcomes	QOL assessment	**√**	**√**	**√**†	n/a	**√**

*Abbreviations:***√** = recommended; **√**ǁ = recommended if clinically indicated; — = not recommended; CBC = complete blood count; CT = computed tomography; MRI = magnetic resonance imaging; n/a = not applicable; PET = positron emission tomography; QOL = quality of life; SFRT = spatially fractionated radiation therapy.

⁎ Weekly.

† Four to 8 weeks after radiation therapy completion.

‡ Routine follow-up every 3 to 4 months in years 1 to 2, every 6 months in years 3 to 5, then yearly.

§ Magnetic resonance imaging preferred, using Response Evaluation Criteria in Solid Tumors and quantitative assessment of tumor necrosis.

¶ Tumor necrosis of at least 90%.

# Feasible weekly or at prospective time points and dose levels during and after treatment.

⁎⁎ Tissue from pretherapy biopsies and postradiation specimens (from the definitive resection) may be procured for correlative studies.

On-treatment evaluations should consist of a standard weekly response, toxicity, QOL assessments, and patient-reported outcomes. Specimen collection of blood and urine multiple times during radiation therapy for translational correlative studies are feasible and strongly recommended (high consensus). Tumor biopsies during the treatment course are considered not clinically feasible (high consensus). Uniquely in the preoperative radiation therapy setting, the surgical specimen after radiation therapy provides an important potential resource for the prospective study of post-SFRT molecular markers in both tumor and normal tissue.

After radiation therapy, preoperative response assessment is recommended, preferably with magnetic resonance imaging, using the Response Evaluation Criteria in Solid Tumors, quantitative imaging assessment of tumor necrosis (>90% necrosis), and standard clinical examination (high consensus) at 4 to 8 weeks after radiation therapy. Evaluations should be performed in conjunction with QOL assessments and patient-reported outcomes (high consensus).

#### Surgical evaluation, pathologic response

Pathologic tumor response, as routinely assessed in standard of care, provides an important outcome assessment for SFRT response in STS clinical trials. Assessment of negative-margin resectability, R0 versus R1 resection, and pathologic criteria of tumor response including quantitative histologic assessment of necrosis of >90%^39^ is required (high consensus).

#### Posttherapy evaluations (after completion of all therapy)

History and clinical examination are indispensable for the assessment of function and toxicity outcomes. Clinical examination and imaging surveillance schedules should follow the standard of care (Table 6) and be combined with patient-reported outcomes and QOL assessments in the routine posttherapy evaluations.

## Conclusion

The pilot experience that has defined SFRT dose and techniques and shown promising tumor control, toxicity outcomes, and early survival outcomes, has reached an inflection point that enables the development of multi-institutional SFRT trials in definitively treated bulky primary tumors.

SFRT for both STS and H&N cancer share common properties of high ablative stereotactic radiation therapy dosing, generally excluding OARs; and are administered in close time proximity before conventional radiation therapy or radiation therapy and chemotherapy.

Standardization of the novel, nonconventional physics and dosimetric parameters with inherent dose and response modeling of the heterogeneous dose to tumor and normal structures is essential for the feasibility of SFRT trials and for their success in generating meaningful results. The inclusion of well-conducted translational science into clinical trial design is critically important to investigate in clinical patients the preclinically observed biological mechanisms and potential immunologic phenomena of SFRT.^15^, ^16^, ^17^, ^18^, ^19^, ^20^ To accomplish this, “liquid biopsy” concepts, leveraged through serial blood and urine collections and synchronized prospectively with the treatment course, may advance our understanding of the underpinnings of SFRT response. The challenge of the inability to procure tumor tissue during the radiation therapy course to interrogate molecular markers may be alleviated by prospective functional and molecular imaging. Finally, physicians’ and physicists’ education was a knowledge gap identified during this consensus effort and is hoped to be addressed by these guidelines.

This development of guidelines for clinical trial design is a novel concept to establish broad consensus (through ample a priori communication and vetting) among the respective scientific and clinical communities, well ahead of clinical trial design and development. We have adapted existing consensus process models that have been in use for clinical care guidelines, which are generally based on ample published data and large numbers of experts. We applied and further developed these concepts for the requirements of consensus development in the different domain of clinical trial design, which has to build on much sparser, less mature pilot data and fewer experts, but nonetheless requires agreement among the broader community to facilitate trial success. Intense engagement and consensus building among clinical, physics, and biology expertise enabled identification of current knowledge gaps and development of design strategies to address them in clinically feasible trials.

The trial design recommendations presented herein are based on the current status of knowledge in SFRT and the developed common understanding among SFRT experts and community. Although they may provide guidance for clinical trial design and embedded translational studies, new data, longer-term outcome results, and larger patient cohorts may further refine, adapt, or modify these initial concepts. Ultimately, these consensus recommendations should be individualized by the respective investigators and their teams pursuing clinical trials in SFRT.

## Appendix Supplementary materials

Image, application 1

Image, application 2

Image, application 3

Image, application 4

Image, application 5

Image, application 6
