# Supplementary material for: An International Consensus on the Design of Prospective Clinical–Translational Trials in Spatially Fractionated Radiation Therapy
Source: Adv Radiat Oncol. 2021 Dec 11;7(2):100866. doi: 10.1016/j.adro.2021.100866 (PMC8843999; doi:10.1016/j.adro.2021.100866)
Supplement: Supplementary file 1 [file mmc1.pdf]

Appendix 1:

Evidence Table - Literature Summary:

SFRT for Head & Neck Cancer

Note:

This collated literature table presents a summary of major pertinent studies that were considered in developing the clinical trial design consensus guideline. The summary table is systematically according to study type, study objective, patient selection, SFRT and conventional radiation therapy parameters, SFRT technology and treatment outcome data.

Abbreviations:

cCR	clinical complete response	pCR	pathologic complete response
cERT	Conventional radiation	PFS	progression-free survival
CR	complete response	PR	partial response
concur	concurrent	pt/pts	patient / patients
DSS	disease-specific survival	RR	response rate
fr	fraction	Tox	toxicity
gr	grade	Tx	treatment
LC	local control	w	with
LR	local recurrence	yr	year
n/a	not applicable		
NR	no response	*	per author's communication
OS	overall survival	–	no data

Studies of Multiple Tumor Sites Including Head and Neck Cancer Patients

Author, Year	Pt No. Sites	Objectives	Methods	Results	Dose/Spatial Fractionation	Conclusion
Mohiuddin M et *al.*<br>(Radiat Oncol Invest 1996; 4:41-7)<br><br>Treated:<br>~1990-1995	61<br><br>72 tumor sites<br><br>GI: 18<br>Sarcoma: 12<br>GU: 9<br>Gyn: 9<br>Melanoma: 5<br>**H&N** (SCCa): **4**<br>Lung: 1<br>Breast: 2<br>Thyroid: 4	Multiple sites<br><br>Palliative only	Study type:<br>Clinical trial<br><br>Study Population:<br>Palliative only tx to refractory, primarily large soft tissue masses.<br>44/72 sites: abdomen/ pelvis<br>24% (17 sites) had prior RT (12.6-79 Gy)<br><br>Outcome Measures:<br>Palliation (pain, bleeding, mass effect): RR, CR, PR, NR<br>Tox (EORTC grading)<br><br>Technique: Block<br><br>Follow-up:<br>median 4 (0.5-28) mo. (d/t advanced stage)<br>10 pts alive ≥ 1 yr	RR: 91%<br><br>LC: Durable response in most pts w longer survival.<br>GRID ≥15 Gy: 100% vs 79% RR for <15 Gy<br>cERT ≥40 Gy: 100% vs 92% RR for <40 Gy<br><br>DSS: –<br><br>OS:<br>27/71 pts: 3-28 mo.<br>10/71 pts: survived >1 yr<br><br>Toxicity:<br>No grade 2 or higher tox.<br>No bowel tox despite 44 pts w abdomen/pelvis tx (1 bowel obstruction due to tumor found at laparotomy)	GRID sequencing:<br>GRID only (32/72 sites).<br>GRID generally first for combined GRID + cERT (in 40/72 sites in pts with life expectancy of >1 mo.)<br><br>GRID method:<br>Block (50% open)<br>6, 24MV<br>Single field<br><br>GRID dose: 10-15/1 (for GRID + cERT)<br>15-25/1 (for GRID only) to Dmax<br><br>cERT dose: (in 40/72) wide range; 78 Gy<br><br>Dose to periphery: –<br><br>OAR dose: –<br><br>Concurr tx: No	GRID therapy results in high (>90%) symptomatic tumor response rate, with minimal toxicity.<br><br>Dose response relationship: High cumulative GRID and cERT doses are needed for satisfactory CR rates:<br>GRID dose ≥15 Gy associated with higher RR, CR;<br>cERT dose ≥40 Gy associated with higher CR.<br><br>**Response by tumor type**:<br>**Best RR** in sarcoma (94%) and **SCCa (92%)**; least RR in adenocarcinoma (69%).<br><br>Parallelism of GRID therapy with brachytherapy, enabling delivery of high doses to small volumes with modest doses over a larger tissue volumes.

Author, Year	Pt No. Sites	Objectives	Methods	Results	Dose/ Spatial Fractionation	Conclusion
Mohiuddin M et *al.*<br>(IJROBP 1999;45:721-7)<br><br><br>Treated:<br>1/1995-3/1998	71<br><br>87 tumor sites<br><br><br>Overall sites:<br>Lung: 18<br>**H&N: 17**<br>Sarcoma:10<br>GI: 4<br>GU: 5<br>Gyn: 8<br>Skin: 11<br>Melan: 3<br>Breast: 3<br>Thyroid: 2<br>Unknown:4<br>Liver: 2	Multiple, palliative 89%<br><br>Curative: +/- sub-sequent surgery 11%	Study type:<br>Retrospective<br><br>Study Population:<br>Palliative: 89% (63/71)<br>Advanced, definitive: H&N/skin ca, 11% (8/71)<br><br>Tumor >8 cm<br><br>Prior RT: 9% (8/87 sites)<br><br>Outcome Measures:<br>RR<br>Pts who died during/within 1 mo. of tx (7) were inevaluable for RR, but were included in toxicity analysis.<br>Path response (available in 8 pts)<br><br>Technique: GRID<br><br>Follow-up:<br>median 7 (3-42) mo.	RR: 76%<br>Palliative pts: 78%<br><br>cCR 63% (5/8 definitive H&N/skin ca pts)<br>cPR 37% (3/8)<br>pCR 4/4 definitive H&N/skin ca pts<br><br>GRID dose ≥15 Gy:<br>RR 94 vs 62% (p=.002)<br><br>cERT dose ≥40 Gy:<br>0 Gy: 86%, 0% (RR, CR)<br><40 Gy: 91%, 13% (RR, CR)<br>≥40 Gy: 94%, 24% (RR, CR)<br><br>LC: –<br><br>DSS: –<br><br>OS: –<br><br>Toxicity:<br>1 gr 3 (mucositis)<br>1 gr 5 (carotid blowout) during tx (rapid tumor lysis)	GRID sequencing:<br>GRID only: 17/71 pts.<br>GRID first, then cERT: 76% (54/71 pts)<br><br>GRID method:<br>Block (50% open)<br>6, 18 MV<br><br>GRID dose: 10-20 Gy/1<br>median: 15 Gy/1 to 10-12 Gy (for prior RT), to Dmax<br><br>cERT dose:<br>Definitive pts (8):<br>50-70 Gy<br>Palliative pts: –<br><br>Dose to periphery: –<br><br>OAR dose: –<br><br>Concurr tx: No	High response, low toxicity.<br><br>Dose response relationship: Validating the results from Mohiuddin et *al.* (Radiat Oncol Invest 1996):<br>GRID dose ≥15 Gy is associated with significantly higher RR.<br>cERT dose ≥40 Gy is associated with higher RR and CR.<br><br>**High RR in definitively treated H&N/skin cancer patients.**<br><br>**Highest CR in SCCa** (29%).

Author, Year	Pt No. Sites	Objectives	Methods	Results	Dose/ Spatial Fractionation	Conclusion
Neuner G et *al.* (IJROBP 2012; 82(5):1642-9)<br><br>Treated: 2003-2008	79<br><br>Lung: 18<br>**H&N: 14**<br>Sarcoma:14<br>Liver: 6<br>Skin: 5<br>Breast: 4<br>Colon/ Anus: 5<br>Kidney: 3<br>Thyroid: 3<br>Esoph: 2<br>Lymph nodes: 2<br>Prostate:1<br>Ovary: 1<br>Unknown:1	Multiple, Palliative 77%, most lung, **H&N**<br><br>Curative: 23%, most lung, **H&N**<br>Pre-op RT 4 pts	Study type: Retrospective<br><br>Study Population: Bulky, median 7.6 cm (4-10 cm)<br>Most lung, H&N, Sarcoma<br><br>Most common tx site: neck<br><br>Outcome Measures: Symptom response:<br>CR= complete resolution<br>PR= any improvement<br>NR= no improvement or progression<br>Imaging response (n=40): per RECIST criteria<br><br>Technique: GRID<br>Retrospective comparison of Block vs. MLC<br><br>Follow-up:<br>2 (0-51.6) mo.<br>28% (22 pts) lost to f/u	RR: Block vs MLC<br>Pain: 75% 74%<br>Mass effect: 67% 73%<br>Bleeding: 50%, 80%<br>Other symptoms: high response.<br><br>Imaging RR (CR+PR):<br>Block vs MLC<br>27% 32%<br><br>LC: –<br><br>DSS: –<br><br>OS: 29% (23/79)<br>(study not intended to report survival)<br><br>Median survival:<br>2.2 mo. (Block)<br>4.1 mo. (MLC), p=NS<br><br>Toxicity:<br>*Early:*<br>2 pts: early gr 4 (skin)<br><br>*Late:*<br>3 pts: late gr 3-4: chronic skin ulceration after cERT dose of 40 Gy, 45 Gy, 60 Gy (2/3 pts with skin involvement)	GRID sequencing:<br>GRID only: 20% (palliative pts)<br>GRID first: 72%, cERT start within 1-2 days<br>In early cERT: 8%<br><br>GRID method:<br>- Block<br>- MLC: average open/ closed ratio 0.31.<br><br>GRID dose: 10-20 Gy (median 15 Gy)/1 fr<br>Block: to Dmax<br>MLC: to GTV, no expansion<br><br>cERT dose: $\geq$35-40 Gy, No dose reduction for GRID, (e.g. 70.2 Gy for H&N), but normal tissue dose reduction<br><br>Dose to periphery: –<br><br>OAR dose: Blocking of neural structures, kidney, GI tract, heart; minimizing exit dose<br><br>Concurr tx:<br>H&N ca/curative:<br>GRID 15 Gy, 1-2 d break, then definitive cERT 70.2 Gy + chemotx (type not reported)	High symptomatic response rate; no difference in response between Block vs MLC based GRID.<br><br>No difference in imaging response for Block vs MLC based GRID therapy.<br><br>Low toxicity rates.<br><br>Ease and efficacy of MLC-based GRID may enable more widespread adoption of SFRT.

Head and Neck Cancer specific Studies						
**Author, Year**	**Pt No. Sites**	**Objectives**	**Methods**	**Results**	**Dose/ Spatial Fractionation**	**Conclusion**
Huhn J et *al.* (Technol Cancer Res Treat 2006; 5:607-12)<br><br>Treated: 7/1995-12/2002	27<br><br>Oral cav: 5<br>Oroph: 14<br>Nasoph: 1<br>Hypoph: 1<br>Unknown:5<br>Oral cav +hypoph: 1	**H&N**, SCCa<br><br>Advanced neck disease<br><br>2 groups: Definitive RT (14)<br>Pre-op RT (13)	Study type: Clinical trial<br><br>Study Population: H&N SCCa with bulky N2-3 neck disease<br><br>*RT only* (14 pts): median tumor size 7 (6-10) cm<br>*Pre-op RT* (13 pts); median 8 (6-13) cm<br><br>Outcome Measures: LC, DSS, Tox<br><br>Technique: GRID<br><br>Follow-up:<br><br>*RT only:* median 10 (3-44) mo.<br>*Pre-op RT:* median 38 (5-116) mo.	RR:<br>*Pre-op RT*: pCR 85%<br><br>LC: Regional/neck:<br>*RT only*: 93% (13/14)<br>*Pre-op RT*: 92% (12/13)<br><br>LC (overall):<br>*RT only*: 86%<br>*Pre-op RT*: 92% (12/13)<br><br>DSS: (3-yr)<br>RT: 50%<br>Pre-op RT: 85%<br><br>OS:<br>*RT only*: 21% (3/14)<br>*Pre-op RT*: 62% (8/13) at 116 mo.<br><br>Toxicity:<br>*RT only*:<br>Early:<br>gr 2-3 skin (number not reported)<br><br>Late:<br>no gr 3<br><br>*Pre-op RT*:<br>3 wound healing complications	GRID sequencing: GRID first<br><br>GRID method: Block<br><br>GRID dose: 15/1;<br>1 pt with 20 Gy/1 fr to Dmax<br>To neck disease only, GRID field off cord<br><br>cERT dose:<br>*RT only/definitive pts* (8): median 70 (68-79) Gy<br>*RT/non-definitive* (6): median 59 (54-60) Gy<br>*Preop RT*: median 59.4 (54-72) Gy<br><br>Hyper- or accelerated fractionation/con-comitant boost: 7/27<br><br>Dose to periphery: –<br><br>OAR dose: Cord excluded from GRID tx<br><br>Concurr tx: during cERT: 7/27 (type not reported)	Very high complete pathologic response rate (85%) for locally advanced neck involvement from H&N cancer. First study to assess pathologic response to GRID and its impact on subsequent local control.<br><br>High regional control in the neck with pre-op and definitive radiation. High survival in preoperative group.<br><br>Surgical approach was feasible and required no alteration.<br><br>Manageable toxicity, including would healing complications.

Author, Year	Pt No. Sites	Objectives	Methods	Results	Dose/ Spatial Fractionation	Conclusion
Penagaricano J et *al.*<br>(IJROBP 2010;76:1369-75)<br><br>Treated:<br>2005-2007*	14<br><br>Tonsil: 4<br>Retromol trigone: 1<br>Base of tongue: 3<br>Larynx: 2<br>Nasoph: 1<br>Maxillary sinus: 2<br>Parotid: 1	**H&N**, SCCa<br><br>Definitive RT, concurrent chemo-therapy<br><br>Tx to lymph nodes: 8 pts, to primary site: 6 pts	Study type:<br>Clinical trial<br><br>Study Population:<br>Bulky H&N ca, >6 cm<br><br>Outcome Measures:<br>LC, DSS, OS,<br>Tox (RTOG criteria)<br><br>Technique:<br>GRID<br><br>Follow-up:<br>median 19 (2-38) mo.<br>10/14 pts had f/u >1 yr	RR:<br>pCR in 8/10 pts (per resection pathology or biopsy of GRID volume)<br><br>LC: crude 79% (11/14)<br>in-field control;<br>no local recurrence in GRID volume<br><br>DSS: crude 79% (11/14)<br><br>OS: 10/14<br>(1 patient from death of disease)<br><br>Toxicity:<br>Early:<br>gr 3 (skin) 7/14<br>gr 3 (mucosal): 4/14<br><br>Late:<br>gr 3 (fibrosis): 1/14<br>PEG dependent trismus (2/14)<br>1 gr 5 carotid blowout after neck dissection at 10 mo. (1/14)<br>Xerostomia: –	GRID sequencing:<br>First, cERT next day<br><br>GRID method:<br>MLC, ~50% open<br><br>GRID dose: 20 Gy/1 fr to Grid-GTV (=bulky, ≥6 cm disease, primary or nodes with no expansion)<br><br>cERT dose: SIB-IMRT/ simultaneous integrated boost (SIB): 66 Gy/30 fr.<br>PTV: 66 Gy<br>Intermediate-risk PTV: 60 Gy<br>Low-risk PTV: 54 Gy<br><br>Dose to periphery: –<br><br>OAR dose:<br>Exclusion of spinal cord, brain stem<br><br>Concurr tx:<br>Full-dose chemotx, most Carboplatin/Docetaxel; 1 pt with 5FU<br>Chemotherapy started on the day of GRID fr	Uniformly treated cohort, all with concurrent chemotx. Chemotherapy also given; chemotherapy also during GRID fraction.<br><br>High response rate, pathologic complete response and local control with no local recurrence within the treated GRID volume.<br><br>Higher early skin toxicity. One gr 5 carotid complication.<br><br>Mucosal toxicity similar to chemotherapy/IMRT series.

Author, Year	Pt No. Sites	Objectives	Methods	Results	Dose/ Spatial Fractionation	Conclusion
Choi JI et *al.*<br><br>(Cureus 2019; ;11(5):e4637. doi: 10.7759/ cureus.4637)<br><br>Treated:<br>2007-2015	21<br><br>(primary sites not reported)	**H&N**, SCCa<br><br>Definitive intent (9)<br>Palliative intent (12)<br><br>9 definite pts: definitive RT, concurr chemo-therapy	Study type:<br>Retrospective<br><br>Study Population:<br>Bulky H&N ca, >5 cm; 5-25 cm, median 9.5 cm<br><br>*Definitive*:<br>all SCCa<br><br>*Palliative*:<br>most SCCa<br><br>Outcome Measures:<br>RR, symptom response, Tox (RTOG criteria)<br><br>Technique:<br>GRID<br><br>Follow-up:<br>7 (4-16) mo.<br>(definitive pts)	RR:<br>*Definitive**:<br>CR: 4/9<br>PR: 1/9<br>Symptom response 8/9:<br>* 1 pt completed only 1 cERT fraction<br><br>*Palliative*:<br>CR: 0/12<br>PR: 5/12<br>Symptom response 6/12<br><br>If received <75% of dose, only 25% symptom response.<br><br>LC: –<br><br>DSS: –<br><br>OS:<br>*Definitive:*<br>7/9 alive at median 7 mo.<br><br>Toxicity:<br>Skin: gr 3: 1/21<br>gr 4: 4/21<br><br>Bleeding: 3/21<br>(2 required hospitalization)<br><br>No gr ≥3mucous membrane tox	GRID sequencing:<br>First, cERT start 1-3 d after GRID<br><br>GRID method:<br>MLC (before 2008)<br>Block (2009-2015)<br><br>GRID dose: 15 Gy/1<br>20 Gy/1 in 5 pts (2010-11)<br><br>cERT dose:<br>*Definitive*: IMRT 69.96 - 72.08 Gy at 2.12 Gy/ fr<br>*Palliative*: 25 Gy/ 10 fr - 78 Gy/39 fr<br><br>Dose to periphery: –<br><br>OAR dose:<br>Maximal avoidance of mandible, spinal cord, brainstem, brain, brachioplexus. Travers-ing smallest possible skin to GTV separation.<br><br>Concurr tx:<br>In all definitive pts.<br>Start after the GRID fr.<br>Cisplatin (9), Cispl+Taxol or Etoposide (5), Cetuximab (2)	SFRT feasible in definitive and palliative setting for large H&N tumors.<br><br>Excellent clinical response with SFRT, cERT and chemotherapy.<br><br>Treatment toxicity acceptable.<br><br>Need for careful patient selection to identify patients who tolerate a full course cERT following SFRT.
