# Supplementary material for: An International Consensus on the Design of Prospective Clinical–Translational Trials in Spatially Fractionated Radiation Therapy
Source: Adv Radiat Oncol. 2021 Dec 11;7(2):100866. doi: 10.1016/j.adro.2021.100866 (PMC8843999; doi:10.1016/j.adro.2021.100866)
Supplement: Supplementary file 2 [file mmc2.pdf]

Appendix 2:

Evidence Table - Literature Summary:

SFRT for Sarcoma

Note:

This collated literature table presents a summary of major pertinent studies that were considered in developing the clinical trial design consensus guideline. The summary table is systematically according to study type, study objective, patient selection, SFRT and conventional radiation therapy parameters, SFRT technology and treatment outcome data.

Abbreviations:

cCR	clinical complete response	pCR	pathologic complete response
cERT	Conventional radiation	PFS	progression-free survival
CR	complete response	PR	partial response
concur	concurrent	pt/pts	patient / patients
DSS	disease-specific survival	RR	response rate
fr	fraction	Tox	toxicity
gr	grade	Tx	treatment
LC	local control	w	with
LR	local recurrence	yr	year
n/a	not applicable		
NR	no response	*	per author's communication
OS	overall survival	–	no data

Multiple-site Studies including Sarcoma Patients

Author, Year	Pt No. Sites	Objectives	Methods	Results	Dose/ Spatial Fractionation	Conclusion
Mohiuddin M et *al.*<br>(Radiat Oncol Invest 1996; 4:41-7)<br><br>Treated:<br>~1990-1995	61<br><br>72 sites	Multiple, Palliative only:<br><br>GI (18)<br>**Sarcoma (12)**<br>GU (9)<br>Gyn (9)<br>Melan (5)<br>ENT(SCCa) (4)<br>Lung (1)<br>Breast (2)<br>Thyroid (4)	Study type:<br>Clinical trial<br><br>Study Population:<br>Palliative only tx to refractory, primarily large soft tissue masses.<br>44/72 sites: abdomen/pelvis<br>24% (17 sites) had prior RT (12.6-79 Gy)<br><br>Outcome Measures:<br>Palliation (pain, bleeding, mass effect): RR, CR, PR, NR<br>Tox (EORTC grading)<br><br>Technique: Block<br><br>Follow-up:<br>median 4 (0.5-28) mo. (d/t advanced stage)<br>10 pts alive ≥ 1 yr	RR: 91%<br><br>LC: Durable response in most pts w longer survival.<br>GRID ≥15 Gy: 100%<br>vs 79% RR for <15 Gy<br>cERT ≥40 Gy: 100%<br>vs 92% RR for <40 Gy<br><br>DSS: –<br><br>OS:<br>27/71 pts: 3-28 mo.<br>10/71 pts: survived >1 yr<br><br>Toxicity:<br>No grade 2 or higher tox.<br>No bowel tox despite 44 pts w abdomen/pelvis tx (1 bowel obstruction due to tumor found at laparotomy)	GRID sequencing:<br>GRID only (32/42 sites).<br>GRID generally first for combined GRID + cERT (in 40/72 sites in pts with life expectancy of >1 mo.)<br><br>GRID method:<br>Block (50% open)<br>6, 24MV<br>Single field<br><br>GRID dose: 10-15/1 (for GRID + cERT)<br>15-25/1 (for GRID only) to Dmax<br><br>cERT dose: (in 40/72) wide range; 78 Gy<br><br>Dose to periphery: –<br><br>OAR dose: –<br><br>Concurr tx: No	GRID therapy results in high (>90%) symptomatic tumor response rate, with minimal toxicity.<br><br>Dose response relationship: High cumulative GRID and cERT doses are needed for satisfactory CR rates:<br>GRID dose ≥15 Gy associated with higher RR, CR;<br>cERT dose ≥40 Gy associated with higher RR, CR.<br><br>**Response by tumor type**:<br>**Best RR in sarcoma (94%)** and SCCa (92%); least RR in adenocarcinoma (69%).<br><br>Parallelism of GRID therapy with brachytherapy, enabling delivery of high doses to small volumes with modest doses over a larger tissue volumes.

Author, Year	Pt No. Sites	Objectives	Methods	Results	Dose/ Spatial Fractionation	Conclusion
Mohiuddin M et *al.*<br>(IJROBP 1999;45:721-7)<br><br>Treated:<br>1/1995-3/1998	71<br><br>87 sites<br><br>Overall sites:<br>Lung: 18<br>H&N: 17<br>**Sarcoma: 10**<br>GI: 4<br>GU: 5<br>Gyn: 8<br>Skin: 11<br>Melan: 3<br>Breast: 3<br>Thyroid: 2<br>Unknown: 4<br>Liver: 2	Multiple, palliative 89%<br><br>Curative: +/- subsequent surgery 11%	Study type:<br>Retrospective<br><br>Study Population:<br>Palliative: 89% (63/71)<br>Advanced, definitive: H&N/skin ca, 11% (8/71)<br><br>Tumor >8 cm<br><br>Prior RT: 9% (8/87 sites)<br><br>Outcome Measures:<br>RR<br>Pts who died during / within 1 mo. of tx (7) were inevaluable for RR, but were included in tox analysis.<br>Path response (available in 8 pts)<br><br>Technique: GRID<br><br>Follow-up:<br>median 7 (3-42) mo.	RR: 76%<br>Palliative pts: 78%<br><br>cCR 63% (5/8 definitive H&N/skin ca pts)<br>cPR 38% (3/8)<br>pCR 50% (4/4 definitive H&N/skin ca pts)<br><br>GRID dose ≥15 Gy:<br>RR 94 vs 62% (p=.002)<br><br>cERT dose ≥40 Gy:<br>0 Gy: 86%, 0% (RR, CR)<br><40 Gy: 91%, 13% (RR, CR)<br>≥40 Gy: 94%, 24% (RR, CR)<br><br>LC: –<br><br>DSS: –<br><br>OS: –<br><br>Toxicity:<br>1 gr 3 (mucositis)<br>1 gr 5 (carotid blowout) during tx (rapid tumor lysis)	GRID sequencing:<br>GRID only: 17/71 pts.<br>GRID first, then cERT: 76% (54/71 pts)<br><br>GRID method:<br>Block (50% open)<br>6, 18 MV<br><br>GRID dose: 10-20 Gy/1 median: 15 Gy/1 to 10-12 Gy (for prior RT), to Dmax<br><br>cERT dose:<br>Definitive pts (8):<br>50-70 Gy<br>Palliative pts: –<br><br>Dose to periphery: –<br><br>OAR dose: –<br><br>Concurr tx: No	High response, low toxicity.<br><br>Dose response relationship: Validating the results from Mohiuddin et *al.* (Radiat Oncol Invest 1996):<br>GRID dose ≥15 Gy is associated with significantly higher RR.<br><br>cERT dose ≥40 Gy is associated with higher RR and CR.<br><br>**Sarcoma (11% of patients) had worse RR, potentially related to the prevalence of very large (>20 cm) tumors and widely disseminated disease.**

Sarcoma specific Studies

Author, Year	Pt No. Sites	Objectives	Methods	Results	Dose/ Spatial Fractionation	Conclusion
Mohiuddin, M et *al.*<br>(IJROBP 2009; 75:S526)<br>(abstr)<br><br>Treated:<br>~before 2009	33<br><br>44 tumor sites:<br><br>Abdomen: 20<br>H&N: 3<br>Chest: 7<br>Extremities: 15	Soft tissue sarcoma<br><br>Definitive/ palliative RT<br><br>Recurrent, unresect-able	<u>Study type:</u><br>Retrospective<br><br><u>Study Population:</u><br>Bulky, median 13 (6-32 cm), unresectable and/or recurrent,<br><br><u>Outcome Measures:</u><br>cRR, pRR, LC, OS<br><br><u>Technique:</u><br>GRID<br><br><u>Follow-up:</u> –<br>for survival median: 9 (2- 44) mo.	<u>RR:</u><br>CR: 26%<br>PR: 50%<br><br>cERT >50 Gy:<br>RR 95%, CR 45%<br><br>cERT<50 Gy:<br>RR 59%, CR 10%<br><br><u>LC:</u> –<br><br><u>DSS</u>: –<br><br><u>OS:</u><br>9/33 alive >1 year<br><br><u>Toxicity:</u><br>Early:<br>gr 3 (skin): 2/33<br><br>Late:<br>no late reactions	<u>GRID sequencing:</u><br>GRID first<br>(4 pts: GRID alone)<br><br><u>GRID method:</u><br>Block<br><br><u>GRID dose:</u> 12-20 Gy (median 15 Gy)/1 fr<br>6 MV<br>to Dmax (6MV)<br><br><u>cERT dose:</u> 22-70 Gy (median 50 Gy)<br><br><u>Dose to periphery:</u> –<br><br><u>OAR dose:</u> –<br><br><u>Concurr tx:</u> No	High response and local control in unresectable and recurrent soft tissue sarcoma, and encouraging survival outcomes (9/33 patients alive >1 year).<br><br>Response higher (95% vs 59%) with cERT dose of >50 Gy.

Author, Year	Pt No.<br>Sites	Objectives	Methods	Results	Dose/ Spatial Fractionation	Conclusion
Mohiuddin, M et *al.*<br>(J Clin Oncol 2014;32: 10575)<br>(abstr)<br><br>Treated:<br>~before 2014	14<br><br>Soft tissue sarcoma<br><br>Sites:<br>Extremities	Curative<br><br>Neo-adjuvant, pre-op RT/concurr chemo-therapy	Study type:<br>Clinical trial<br><br>Study Population:<br>Locally advanced soft tissue sarcoma, ≥8 cm, median 11.5 cm (8-26 cm)<br><br>Outcome Measures:<br>Tumor necrosis (>90%)<br>Resectability<br>Limb salvage<br>LC<br>DSS, OS<br><br>Technique:<br>GRID<br><br>Follow-up:<br>median 14 (3-43) mo.	RR:<br>>90 % tu necrosis: 64%<br>>80% tu necrosis: 2/14<br>pCR: 2/14<br><br>Limb salvage surgery:<br>93% (13/14)<br><br>LC: 100%<br><br>DSS:<br>86%: 12/14 alive/NED<br><br>OS:<br>86%: 12/14 alive/NED<br><br>Toxicity:<br>1 gr 3 skin reaction resulting in tx interruption/dis-continuation<br><br>2 delayed wound healing	GRID sequencing:<br>GRID first<br><br>GRID method:<br>Block<br><br>GRID dose: 18 Gy/1<br><br>cERT dose: 50 Gy<br><br>Dose to periphery: –<br><br>OAR dose: [illegible]<br><br>Concurr tx:<br>Iphosfamide/Mesna every 3 weeks	Induction SFRT/GRID therapy and conventional ERT with chemotherapy enhances necrosis and response to neoadjuvant chemo/radiation therapy, and potentially improves local control, limb salvage and survival in locally advanced extremity sarcoma.

Author, Year	Pt No. Sites	Objectives	Methods	Results	Dose/ Spatial Fractionation	Conclusion
Snider, JW<br>et *al.*<br><br>Rad Res 2020; 194: 707-14<br><br><br>Treated:<br>2005-2019	26<br><br>Soft tissue sarcoma: 21<br><br>Lower extremity: 12<br>Upper extremity: 3<br>Pelvis: 3<br>Trunk: 3<br>Retroperit: 3<br>Abdomen: 1<br>H&N: 1<br><br><br><br>Undiff pleomorphic: 7<br>Liposarc: 4<br>Myxofibrosarc: 3<br>Extraoss. osteosarc: 4<br>Chondrosarcoma: 1<br>Other: 7	Curative:<br>Neo-adjuvant, pre-op RT<br><br><br>Prior tx:<br>Resection (followed by progression):3<br><br>Chemotherapy (followed by progression):4	Study type:<br>Retrospective<br><br>Study Population:<br>Locally advanced sarcoma, all except 2 pts > 10 cm, median 14.2 cm (8.8-40 cm)<br><br><br>Grade 2-3: 88%<br>Grade 1: 3/26<br><br>Outcome Measures:<br>pCR, LC, PFS, OS, Tox<br><br>Technique:<br>GRID<br><br>Follow-up:<br>median 25 (7-109*) mo.	RR:<br>pCR (>80% necrosis): 32%<br>Hi gr soft tissue sarcoma: 35% (6/17) pCR<br>Hi gr extremity sarcoma: 50% (4/8) pCR<br><br>pCR poor in low grade tumors<br><br>Negative resection margins: 77% (20/26), close margins common<br><br>R0/R1 resection in all pts.<br>pathologic stage:<br>ypT2a (3 pts)<br>ypT2b (23 pts)<br><br>LC (2-yr):<br>85%<br><br>PFS (2-yr):<br>65%<br><br>OS (2-yr):<br>86%<br><br>Toxicity:<br>35% major wound complications (per NCIC definition)	GRID sequencing:<br>GRID first<br>2-3 day break between GRID and cERT<br><br>GRID method:<br>Block, MLC<br>Image guidance<br><br>GRID dose: 15 Gy /1<br><br>cERT dose: 45-50.4 Gy at 1.8-2.25 Gy/fr (protons in 2 pts), no boost<br><br>Dose to periphery: –<br><br>OAR dose: –<br>Avoiding critical organs<br><br>Concurr tx:<br>No<br>(prior chemotherapy followed by progression: 4 pts)	SFRT with GRID therapy, followed by conventional ERT is a safe and effective neoadjuvant regimen for high-risk bulky soft tissue sarcomas.<br><br>The pathologic response rate of 35% exceeds that of conventionally fractionated RT alone (19.4% in RTOG 0630) and RT/chemotherapy (27.5% in RTOG 9514) despite the larger tumor size in the current series. Toxicity is acceptable and lower than in the NCIC trial.
