# Supplementary material for: An International Consensus on the Design of Prospective Clinical–Translational Trials in Spatially Fractionated Radiation Therapy
Source: Adv Radiat Oncol. 2021 Dec 11;7(2):100866. doi: 10.1016/j.adro.2021.100866 (PMC8843999; doi:10.1016/j.adro.2021.100866)

Appendix 3:

SFRT Clinical Trial Consensus for Head and Neck Cancer
Aggregated Voting Round 1 and 2 Results and Expert Panel Consensus

Voting rank and Voting categories are 1,2,3=not appropriate -- 4,5,6=may be appropriate -- 7,8,9=appropriate

Column **Vote category** denotes the vote category (*Appr* = Appropriate, *May be appr* = May be appropriate, *Not* appr = Not appropriate) that received the highest percentage of votes. The percentage of agreement with the vote category is shown in column **%Agreement with Vote**.

Column **Agreement** denotes level of agreement based on Voting Round 1 and Voting Round 2 (shown as "V2" in left column): *high*, *moderate* or *low* as defined in the table below:

Agreement	Definition
High	Percent agreement ≥ 67% AND if any disagreement, it is by at most 1 voting category
Moderate	60-67% agreement OR agreement ≥67% but votes in both *Appropriate* and *Not appropriate* vote categories
Low	Percent agreement <60%

Column **Expert Panel Consensus** denotes resulting consensus (*high*, *moderate* or *low*) based on the Expert Panel's review of the voting rounds and deliberation.

Column **Expert Panel Conclusions** denotes additional conclusions by the Expert Panel not contained in the criteria and consensus results.

Abbreviations:
- Nr = number
- Exp. Panel = Expert Panel
- RT = radiation therapy
- V2 = voting round 2

	Criteria	Vote 1 and 2								Expert Panel	
		Nr of Votes	Min Rank	Max Rank	Range of ranks	Mean Rank	%Agreement with Vote Category	Vote Category	Agreement	Exp. Panel Consensus	Exp. Panel Conclusions
1	**Eligible Disease Sites:**										
	Oropharynx	5	4	9	5	7.2	80%	Appr	high	high	There is insufficient meaningful clinical data to make the case to favor a particular H&N primary site. > *Decision for 2nd voting round (see V2).*
	Hypopharynx	5	4	9	5	6.6	60%	Appr	moderate	high	
	Supraglottic Larynx	5	4	8	4	5.6	60%	May be appr	moderate	high	
	Larynx	5	4	8	4	5.6	60%	May be appr	moderate	high	
	Nasopharynx	5	4	9	5	6.4	60%	Appr	moderate	moderate	
V2	While oro-, hypo-, nasopharynx, supraglottic and glottic larynx tumors are eligible, there is insufficient clinical evidence in favor of specific H&N primary sites for SFRT trials	5	5	9	4	7.6	80%	Appr	high	high	Agreed.

Rating scale and Vote categories: 1,2,3=not appropriate -- 4,5,6=may be appropriate -- 7,8,9=appropriate

		Vote 1 and 2								Expert Panel	
	**Criteria**	**Nr of Votes**	**Min Rank**	**Max Rank**	**Range of ranks**	**Mean Rank**	**%Agreement with Vote Category**	**Vote Category**	**Agreement**	**Exp. Panel Consensus**	**Exp. Panel Conclusions**
2	**Eligible Disease Stage, Tumor Size (largest diameter clinically or by imaging):**										
	T3-4 and/or N3	5	7	9	2	7.8	100%	Appr	high	N/A	<u>Panel disagrees.</u> Eligibility should be based on bulky lymph node status (N3, >6 cm), not the primary tumor. Safety of GRID therapy to *primary* tumor sites is not sufficiently established (high consensus). > *Decision for 2nd voting round (see V2).*
	Tumor and/or Lymph node size >6 cm	5	7	9	2	8.0	100%	Appr	high		
V2	N3 with any T-stage	5	9	9	0	9.0	100%	Appr	high	high	Agreed.
	Single node or matted lymph nodes, totaling >6 cm	5	7	9	2	8.2	100%	Appr	high	high	Agreed.

		Vote 1 and 2								Expert Panel	
	**Criteria**	**Nr of Votes**	**Min Rank**	**Max Rank**	**Range of ranks**	**Mean Rank**	**%Agreement with Vote Category**	**Vote Category**	**Agreement**	**Exp. Panel Consensus**	**Exp. Panel Conclusions**
3	**Eligible Histology, and Tumor Markers:**										
	Squamous cell ca.	5	7	9	2	8.2	100%	Appr	high	high	Agreed.
	HPV negative	5	7	9	2	8.2	100%	Appr	high	high	Inclusion of HPV-positive tumors controversial; > *decision for 2nd voting round (V2).*
V2	Should HPV-positive patients with N3 neck disease be included?	5	3	9	6	5.8	40%	Appr May be appr	low	high	Inclusion of patients with bulky HPV-positive neck nodes may be appropriate.
	Other histology/Tumor markers:	1								high	Radiosensitive and unusual histologies are excluded.
	Comment: Exclude radiosensitive tumors (e.g. lymphoma), sarcomas, adeno-ca, and undifferentiated tumors										

Rating scale and Vote categories: 1,2,3=not appropriate -- 4,5,6=may be appropriate -- 7,8,9=appropriate

		Vote 1 and 2								Expert Panel	
	**Criteria**	**Nr of Votes**	**Min Rank**	**Max Rank**	**Range of ranks**	**Mean Rank**	**%Agreement with Vote Category**	**Vote Category**	**Agreement**	**Exp. Panel Consensus**	**Exp. Panel Conclusions**
4	**Eligible Age:**										
	>18 years old	5	7	9	2	8.2	100%	Appr	high	high	
	<85 years old	5	3	9	6	6.8	60%	Appr	moderate	N/A	Contradictory results; *> decision for 2nd voting round.*
	No upper age limit	4	4	9	5	7.0	75%	Appr	high	N/A	
	Comments:<br>1. Consider child with large tumor.<br>2. <15 years; > 85 years with good KPS	2									
V2	Is it appropriate to include patients >85 years (with acceptable performance status) into SFRT trials?	5	6	9	3	7.4	80%	Appr	high	high	No upper age limit if eligible based on performance status.
	Comments:<br>1. Depends on endpoint<br>2. KPS with life expectancy of at least 5 years.	2									

		Vote 1 and 2								Expert Panel	
	**Criteria**	**Nr of Votes**	**Min Rank**	**Max Rank**	**Range of ranks**	**Mean Rank**	**%Agreement with Vote Category**	**Vote Category**	**Agreement**	**Exp. Panel Consensus**	**Exp. Panel Conclusions**
5	**Stratifications:**										
	T-stage	5	3	9	6	6.4	60%	Appr	moderate	high	Stratification can be considered but has to be weighed with the ensuing need for increased sample size.
	Comments:<br>T3/T4; T3-T4	2									
V2	T-stage	5	4	8	4	5.2	80%	May be appr	high		
	N-stage	5	4	9	5	7.4	80%	Appr	high	N/A	Panel disagrees. N-stage is the eligibility criterion (see #2) (high consensus).
	Comments:<br>1. N3; T1 large neck mass<br>2. Oncologist preference	2									
	HPV status	4	4	9	5	7.3	75%	Appr	high	high	Agreed based on the strong effect of HPV status on prognosis.
	SFRT technology	5	5	9	4	7.2	80%	Appr	high	high	Agreed based on the variability of dose distribution with different GRID technologies and Lattice therapy (if Lattice therapy is used).
	Systemic therapy	4	3	8	5	6.3	50%	Appr	low	high	Stratification according to systemic therapy, which is commonly used, is not necessary.

	Criteria	Vote 1 and 2								Expert Panel	
		Nr of Votes	Min Rank	Max Rank	Range of ranks	Mean Rank	%Agreement with Vote Category	Vote Category	Agreement	Exp. Panel Consensus	Exp. Panel Conclusions
6	**EXCLUSION Criteria: These disease sites should be EXCLUDED from an SFRT trial:**										
	Exclude Salivary gland tumors	5	1	8	7	4.2	40%	Not appr<br>May be appr	low	high	Exclude salivary gland and paranasal sinus tumors due to rare primary site and variability in histology that can add confounding variables to a trial.
	Exclude Paranasal sinus tumors	5	1	8	7	4.0	40%	Not appr<br>May be appr	low	high	
	Exclude Primary skin cancer	5	1	8	7	3.8	60%	Not appr	moderate	high	Patients with primary skin cancer may be included if they are eligible according to N3 lymph node status.
	Comment:<br>1. Include as long as inoperable, meets size requirements.<br>2. Large-volume bulky tumors and regional nodes can be treated.	2									
	Other Exclusions	2								high	Radiosensitive and unusual histologies are excluded.
	Comments:<br>1. Tumors that are considered sensitive such as leukemia, lymphoma, multiple myeloma, etc.;<br>2. Lymphomas and or bulky leukemic infiltrates										

Rating scale and Vote categories: 1,2,3=not appropriate -- 4,5,6=may be appropriate -- 7,8,9=appropriate

		Vote 1 and 2								Expert Panel	
	**Criteria**	**Nr of Votes**	**Min Rank**	**Max Rank**	**Range of ranks**	**Mean Rank**	**%Agreement with Vote Category**	**Vote Category**	**Agreement**	**Exp. Panel Consensus**	**Exp. Panel Conclusions**
7	**EXCLUSION Criteria: These disease sites should be EXCLUDED from an SFRT trial** (con't):										
	Recurrent tumors (after prior radiation)	5	1	6	5	3.8	60%	May be appr	moderate	moderate	Previous radiation is excluded. For previously irradiated patients a separate subsequent trial should be developed.
	Recurrent tumors (after prior surgery)	5	1	7	6	3.6	60%	Not appr	moderate	N/A	Moderate consensus of voters and Panel for inappropriateness to exclude; > *decision for 2nd voting round.*
V2	Are patients with recurrent bulky (N3) neck disease after prior surgery (if not previously irradiated) eligible?	5	9	9	0	9.0	100%	Appr	high	high	Patients with recurrent bulky (N3) neck disease after prior surgery (if not previously irradiated) do NOT need to be excluded.
	Carotid artery invasion	5	2	9	7	5.6	40%	Appr<br>May be appr	low	high	Exclude patients with *both* carotid invasion and skin involvement and patients with *both* carotid invasion and re-irradiation (based on 2 cases of carotid blowout: 1 with skin involvement , 1 with re-irradiation).
	Scleroderma (Systemic sclerosis)	5	3	9	6	5.0	60%	May be appr	moderate	N/A	Moderate consensus among voters, inconclusive Panel discussion; >*decision for 2nd voting round.*
V2	Patients with Scleroderma (systemic sclerosis) should be EXCLUDED	5	4	9	5	7.0	80%	Appr	high	high	Patients with active scleroderma should be excluded.

		Vote 1 and 2								Expert Panel	
	**Criteria**	**Nr of Votes**	**Min Rank**	**Max Rank**	**Range of ranks**	**Mean Rank**	**%Agreement with Vote Category**	**Vote Category**	**Agreement**	**Exp. Panel Consensus**	**Exp. Panel Conclusions**
8	**Pre-treatment Evaluations: These clinical and histologic investigations should be required:**										
	CT Maxillo/facial/neck	4	8	9	1	8.5	100%	Appr	high	high	Agreed.
	MRI Maxillo/facial/neck	5	5	9	4	7.4	80%	Appr	high	high	Agreed.
	CT Chest	5	2	9	7	6.8	80%	Appr	moderate	high	Chest with upper abdomen (liver).
	CT Chest/abdomen	5	2	8	6	5.4	40%	Appr<br>May be appr	low		
	PET/CT	4	6	9	3	7.8	75%	Appr	high	high	Agreed.
	Fiberoptic laryngoscopy	5	5	8	3	6.2	60%	May be appr	moderate		Agreed, to be used as indicated.
	Swallowing study	5	5	6	1	5.6	100%	May be appr	high	high	Agreed.
9	HPV testing	5	5	9	4	7.6	80%	Appr	high	high	Agreed.
	Other evaluations:	2								high	Overall, pre-treatment evaluations follow the standard of care.
	Comment:<br>CBC, CMP										

	Criteria	Vote 1 and 2								Expert Panel	
		Nr of Votes	Min Rank	Max Rank	Range of ranks	Mean Rank	%Agreement with Vote Category	Vote Category	Agreement	Exp. Panel Consensus	Exp. Panel Conclusions
10	**Radiation Therapy: SFRT Dose:**										
	15 Gy in 1 fraction	5	1	8	7	6.0	60%	Appr	moderate	high	A dose of 15 Gy in 1 fraction is recommended based on existing outcome studies. Effects from the widely used concurrent chemotherapy (that was not used in all prior studies) has to be considered. A dose of 20 Gy/1 fraction has been largely used for palliative treatment. SFRT should be given in no more than 1 fraction.<br><br>The EUD must be defined for dose prescription because of differing dose distributions for different GRID technologies/ techniques and Lattice therapy (if Lattice therapy is used in the future).
	18 Gy in 1 fraction	5	1	8	7	6.0	60%	Appr	moderate		
	20 Gy in 1 fraction	5	1	8	7	5.2	40%	Appr<br>May be appr	low		
	15-18 Gy in 1 fraction	5	4	9	5	7.2	60%	Appr	moderate		
	Other dose:	2									
	Comments:<br>1. 15 or 18 Gy/1 fraction<br>2. 3 fractions > 8-10 Gy/ fraction in vertices										

		Vote 1 and 2								Expert Panel	
	**Criteria**	**Nr of Votes**	**Min Rank**	**Max Rank**	**Range of ranks**	**Mean Rank**	**%Agreement with Vote Category**	**Vote Category**	**Agreement**	**Exp. Panel Consensus**	**Exp. Panel Conclusions**
11	**Radiation Therapy: SFRT Target volume:**										
	GTV (primary or nodal mass) without margin	5	8	9	1	8.4	100%	Appr	high	N/A	Panel disagrees with SFRT to the primary tumor; *> decision for 2nd voting round (V2).*
V2	SFRT should be given to bulky lymph nodes (NOT to the primary tumor)	5	5	9	4	7.8	80%	Appr	high	high	Agreed.
12	**Radiation Therapy: SFRT: Normal Organ-at-Risk structures:**										
	Exclude spinal cord	5	8	9	1	8.6	100%	Appr	high	High	Agreed.
	Exclude brain stem	5	5	9	4	8.0	80%	Appr	high	High	Agreed.
	Exclude optic nerves, chiasm	5	5	9	4	8.0	80%	Appr	high	High	Agreed.
	Exclude brachial plexus	5	3	9	6	7.0	80%	Appr	moderate	moderate	May not always be feasible.
	Addition of PRV margins to normal structures	5	5	9	4	6.4	60%	Maybe appr	moderate	moderate	Moderate consensus; *decision for 2nd voting round.*
V2	Addition of PRV margins to normal structures	5	3	7	4	4.8	60%	May be appr	moderate	moderate	Addition of PRV margins to normal structures may be appropriate, particularly to spinal cord and brainstem.
	Other OARs:	1									Panel disagrees. Carotid is not excluded. See also #7.
	Comment: Exclude carotid										

Rating scale and Vote categories: 1,2,3=not appropriate -- 4,5,6=may be appropriate -- 7,8,9=appropriate

		Vote 1 and 2								Expert Panel	
	**Criteria**	**Nr of Votes**	**Min Rank**	**Max Rank**	**Range of ranks**	**Mean Rank**	**%Agreement with Vote Category**	**Vote Category**	**Agreement**	**Exp. Panel Consensus**	**Exp. Panel Conclusions**
13	**Radiation therapy – SFRT: SFRT technique:**										
	GRID (collimator-based)	5	6	9	3	7.6	80%	Appr	high	high	Agreed.
	GRID (MLC-based)	5	8	9	1	8.2	100%	Appr	high	high	Agreed.
	GRID (either within same trial)	5	8	9	1	8.6	100%	Appr	high	high	Agreed.
	Lattice	4	7	9	2	8.3	100%	Appr	high	high	Agreed. No published experience in Lattice. GRID preferable.
	Other	2								high	Physics parameters of collimator-based and MLC-based GRID must be clearly defined because of the variability in technique (e.g. one vs multiple gantry angles) between collimator -based and MLC-based GRID and Lattice.<br><br>Lattice may become an option in future if more clinical experience in Lattice for H&N cancer emerges.
	Comments:<br>1. stratify arms GRID vs Lattice<br>2. Could stratify based on GRID vs Lattice										

	Criteria	Vote 1 and 2								Expert Panel	
		Nr of Votes	Min Rank	Max Rank	Range of ranks	Mean Rank	%Agreement with Vote Category	Vote Category	Agreement	Exp. Panel Consensus	Exp. Panel Conclusions

14	Radiation therapy – Conventional ERT: Dose and technique:										
	PTV/Primary: 66Gy<br>PTV/High-risk: 60Gy<br>PTV/Low-risk: 50-54Gy	5	4	8	4	6.4	60%	Appr	moderate	high	Doses are acceptable and should be tailored according to primary tumor and extent of involvement.<br><br>EUD of SFRT and conventional ERT must be documented.
	PTV/Primary: 70-72Gy<br>PTV/High-risk: 60-63Gy<br>PTV/Low-risk: 50-56Gy	5	8	9	1	8.4	100%	Appr	high		
	Comment:<br>Based on current practice	1									
	IMRT	5	8	9	1	8.4	100%	Appr	high	high	Agreed.
	IMRT with SIB	5	4	9	5	6.8	60%	Appr	moderate	high	SIB acceptable, but an SIB may add additional variability to the treatment regimen.
	Comments:<br>1. adds too many variables;<br>2. If other location of gross disease in H/N besides the bulky lesion	2									

15	Radiation therapy – Conventional ERT: OAR constraints:										
	Conventional constraints without consideration for SFRT dose	5	7	9	2	8.2	100%	Appr	high	high	Agreed.

		Vote 1 and 2								Expert Panel	
	**Criteria**	**Nr of Votes**	**Min Rank**	**Max Rank**	**Range of ranks**	**Mean Rank**	**%Agreement with Vote Category**	**Vote Category**	**Agreement**	**Exp. Panel Consensus**	**Exp. Panel Conclusions**
16	**On-therapy Evaluations: Evaluate feasibility:**										
	Toxicity assessment (weekly)	5	8	9	1	8.6	100%	Appr	high	high	Agreed.
	Correlative studies (blood, urine): pre-RT, 1 x during RT, post-RT	4	5	9	4	7.3	75%	Appr	high	high	Agreed.
	Correlative studies (blood, urine): pre-RT, 3 x during RT, post-RT	5	1	9	8	5.4	40%	Appr<br>May be appr	low	high	Appropriate and feasible. Patients tend to have weekly blood draws for chemotherapy. Biomarker sampling for vascular and inflammatory signals of interest.
	Comment: Blood sample 1-3 days after SFRT, before starting conventional fraction	1									
	Tumor biopsies twice during radiation therapy	4	1	7	6	3.5	75%	Not appr	moderate	high	Tumor biopsies during radiation therapy not clinically feasible. Consider functional/ molecular imaging instead. Biopsies not clinically feasible or implementable
	Comment: Biopsy immediately after SFRT to assess inflamm. response	1									
	Normal tissue biopsies twice during radiation therapy	5	1	5	4	3.4	60%	Not appr	moderate		
	QOL assessment<br>Comment:<br>During weekly visits	5<br>1	7	9	2	8.0	100%	Appr	high	high	Agreed.
	Patient reported outcomes	5	8	9	1	8.6	100%	Appr	high	high	Agreed.
	Comment: Weekly	1									
	Other on-therapy evaluations:	1								high	Agreed. Criteria for adaptive therapy can be established based on CBCT findings.
	Comment: CBCT daily, adaptive therapy at the discretion of rad. oncologist										

		Vote 1 and 2								Expert Panel	
	**Criteria**	**Nr of Votes**	**Min Rank**	**Max Rank**	**Range of ranks**	**Mean Rank**	**%Agreement with Vote Category**	**Vote Category**	**Agreement**	**Exp. Panel Consensus**	**Exp. Panel Conclusions**
17	**Concurrent systemic therapy: Agents:**										
	Cisplatin	5	6	9	3	7.8	80%	Appr	high	high	Any chemotherapy that is considered acceptable and to have acceptable toxicity when given concurrently with standard fractionation RT is allowed.
	Comment: As per current practice	1									
	Taxanes	4	3	8	5	6.0	50%	Appr	low		
	Comment: Depends on the oncologist	1									
	Cetuximab	5	3	8	5	6.2	60%	Appr	moderate		
	Other systemic therapy:	1									
	Comment: Depends on oncologist										

		Vote 1 and 2								Expert Panel	
	**Criteria**	**Nr of Votes**	**Min Rank**	**Max Rank**	**Range of ranks**	**Mean Rank**	**%Agreement with Vote Category**	**Vote Category**	**Agreement**	**Exp. Panel Consensus**	**Exp. Panel Conclusions**
18	**Concurrent systemic therapy: Timing:**										
	Systemic therapy DURING SFRT FRACTION permitted	5	3	9	6	5.6	40%	Appr<br>May be appr	low	high	Not allowed. SFRT without chemotherapy is recommended; then conventionally fractionated ERT with chemotherapy starts 72 hours later (per clinical and published experience). Common schedule: SFRT on a Friday, followed by chemo/radiation therapy start on a Monday.
	Neoadjuvant systemic therapy NOT allowed	5	3	9	6	4.4	60%	Not appr	moderate	high	Neoadjuvant chemotherapy is not allowed.
		1									

		Vote 1 and 2								Expert Panel	
	**Criteria**	**Nr of Votes**	**Min Rank**	**Max Rank**	**Range of ranks**	**Mean Rank**	**%Agreement with Vote Category**	**Vote Category**	**Agreement**	**Exp. Panel Consensus**	**Exp. Panel Conclusions**

19	**Concurrent systemic therapy: Immunotherapy:**										
	Immunotherapy as part of trial regimen	4	3	8	5	6.0	50%	Appr	low	moderate	Panel prefers to not include immunotherapy in an initial trial. There is no published experience with SFRT+ immune-therapy in H&N cancer, although it has been clinically widely used in the general re-irradiation/ palliative setting.<br><br>The combination with immunotherapy is an area of high interest and SFRT/immune-therapy combination should be considered for a separate/subsequent trial.
	Comment:<br>Too many variables	1									

Rating scale and Vote categories: 1,2,3=not appropriate -- 4,5,6=may be appropriate -- 7,8,9=appropriate

	Criteria	Vote 1 and 2								Expert Panel	
		Nr of Votes	Min Rank	Max Rank	Range of ranks	Mean Rank	%Agreement with Vote Category	Vote Category	Agreement	Exp. Panel Consensus	Exp. Panel Conclusions
**20**	**Post-therapy Evaluations: Clinical:**										
	Clinical exam	5	8	9	1	8.4	100%	Appr	high	high	Agreed.
	Fiberoptic exam	4	7	9	2	8.0	100%	Appr	high	high	Agreed.
	Comment: if pertinent	1									
	Toxicity assessment	5	8	9	1	8.6	100%	Appr	high	high	Agreed.
	Every 3 months (year 1-2)	4	4	9	5	7.5	75%	Appr	high	high	Agreed.
	Comment: Once a month 1 year; second year 2-3 months	1									
	Every 4-6 months (year 3-5)	5	8	9	1	8.6	100%	Appr	high	high	Agreed.
	Comment: Every 6 months	1									
	QOL assessment	5	8	9	1	8.6	100%	Appr	high	high	Agreed.
	Patient reported outcomes	4	8	9	1	8.8	100%	Appr	high	high	Agreed.

**21**	**Post-therapy Evaluations: Imaging:**										
	CT Maxillo/facial/neck	5	3	9	6	7.0	80%	Appr	moderate	high	Agreed; appropriate.
	Comment: as appropriate in standard practice	1									
	MRI Maxillo/facial/neck	5	7	9	2	7.8	100%	Appr	high	high	Agreed.
	PET/CT 3 months post-therapy	5	8	9	1	8.4	100%	Appr	high	high	Agreed.

22	Knowledge gaps in H&N cancer	
	Clinical:	Lack of SFRT clinical trials
		Differences and impact of SFRT on systemic and local control outcomes
		Appropriate combined therapy, optimal inclusion of chemotherapy/immunotherapy
		Role of SFRT for primary disease (current data primarily based in treatment of bulky lymph nodes)
		Role of SFRT in patients with moderate bulk of disease
		Tolerance
		Physician education
	Physics:	Treatment dose and number of fractions
		Standardization of SFRT delivery systems
	Biology:	Mechanism of action
		Immunological effects
		Biological cues that can be harnessed for improved outcomes

23	Other comments
	None

24	**Demographics of Voters**

<u>Demographics of voters:</u>

- I am practicing or have practiced clinical SFRT in patient care.
- I am practicing or have practiced clinical SFRT in Head & Neck cancer patients.
- I have presented abstract(s) on clinical SFRT including patient outcomes.
- I have presented abstract(s) on clinical SFRT including patient outcomes in Head & Neck cancer patients.
- I have published scientific article(s) on clinical SFRT including patient outcomes.
- I have published scientific article(s) on clinical SFRT including patient outcomes in Head & Neck cancer patients.

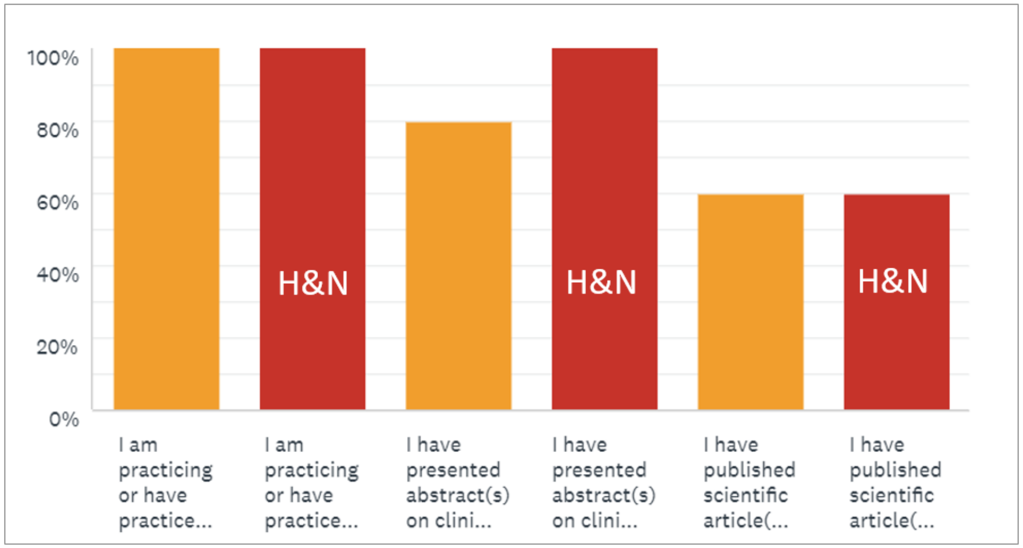

Supplement: Supplementary file 3 [file mmc3.pdf]
