# Supplementary material for: An International Consensus on the Design of Prospective Clinical–Translational Trials in Spatially Fractionated Radiation Therapy
Source: Adv Radiat Oncol. 2021 Dec 11;7(2):100866. doi: 10.1016/j.adro.2021.100866 (PMC8843999; doi:10.1016/j.adro.2021.100866)

Appendix 4:

SFRT Clinical Trial Consensus for Sarcoma
Aggregated Voting Round 1 Results and Expert Panel Consensus

Voting rank and Voting categories are 1,2,3=not appropriate -- 4,5,6=may be appropriate -- 7,8,9=appropriate

Column **Vote category** denotes the vote category (*Appr* = Appropriate, *May be appr* = May be appropriate, *Not* appr = Not appropriate) that received the highest percentage of votes. The percentage of agreement with the vote category is shown in column **%Agreement with Vote**.

Column **Agreement** denotes level of agreement based on the Voting Round 1: *high*, *moderate* or *low* as defined in the table below:

Agreement	Definition
High	Percent agreement $\geq$ 67% AND if any disagreement, it is by at most 1 voting category
Moderate	60-67% agreement OR agreement $\geq$67% but votes in both *Appropriate* and *Not appropriate* vote categories
Low	Percent agreement <60%

Column **Expert Panel Consensus** denotes resulting consensus (*high*, *moderate* or *low*) based on the Expert Panel's review of the voting rounds and deliberation.

Column **Expert Panel Conclusions** denotes additional conclusions by the Expert Panel not contained in the criteria and consensus results.

Abbreviations:
- Nr = number
- Exp. Panel = Expert Panel
- RT = radiation therapy

		Vote 1 and 2								Expert Panel	
	**Criteria**	**Nr of Votes**	**Min Rank**	**Max Rank**	**Range of ranks**	**Mean Rank**	**%Agreement with Vote Category**	**Vote Category**	**Agreement**	**Exp. Panel Consensus**	**Exp. Panel Conclusions**
1		**Eligibility Criteria: Stage and tumor size (largest diameter by imaging)**									
	Stage IB - IIIB	4	5	8	3	7.0	75%	Appr	high	high	Agreed.
	No lymph node involvement	3	5	9	4	6.7	67%	May be appr	high	high	Agreed.
	Tumor size >8 cm	4	9	9	0	9.0	100%	Appr	high	high	Agreed.
	Unresectable tumor	4	4	9	5	7.8	75%	Appr	high	high	Agreed.
	Plan for preoperative radiation	4	8	9	1	8.8	100%	Appr	high	high	Agreed.

		Vote 1 and 2								Expert Panel	
	**Criteria**	**Nr of Votes**	**Min Rank**	**Max Rank**	**Range of ranks**	**Mean Rank**	**%Agreement with Vote Category**	**Vote Category**	**Agreement**	**Exp. Panel Consensus**	**Exp. Panel Conclusions**
2		**Eligible Histologies**									
	Undiff. pleomorphic sacrcoma	4	9	9	0	9.0	100%	Appr	high	high	Agreed.
	Myxoid Liposarcoma	3	6	9	3	7.7	67%	Appr	high		
	Comment: tend to be radiation sensitive tumors	1									
	Leiomyosarcoma	4	9	9	0	9.0	100%	Appr	high	high	Agreed.
	Osteoarcoma	3	4	9	5	7.0	67%	Appr	high	high	Panel disagrees. Extra-skeletal osteosarcoma, which is rare, should be excluded.
	Grade 2-3	4	9	9	0	9.0	100%	Appr	high	high	Agreed.
	Any grade	3	2	9	7	4.3	67%	Not appr	moderate	high	Grade 2 and 3 tumors are eligible due to the poorer prognosis and local control. Inclusion of more aggressive histologies should be considered in a future study. The redundancy in molecular pathways makes molecular targeting in sarcomas difficult. Post-hoc stratification may be done p53, Rb, EWS and non-EWS fusion proteins. However, for an initial study, practical

Rating scale and Vote categories: 1,2,3=not appropriate -- 4,5,6=may be appropriate -- 7,8,9=appropriate

											considerations of recruitment (in view of the rarity of sarcoma), would make sub-classification or stratification based on molecular markers challenging.
	Comment:<br>If it meets other criteria	1								N/A	<u>Panel disagrees</u>.

	Criteria	Vote 1 and 2								Expert Panel	
		Nr of Votes	Min Rank	Max Rank	Range of ranks	Mean Rank	%Agreement with Vote Category	Vote Category	Agreement	Exp. Panel Consensus	Exp. Panel Conclusions
3	**Eligible Age**										
	>18 years old	4	9	9	0	9.0	100%	Appr	high	high	Agreed.
	<85 years old	3	1	7	6	5.0	67%	Appr	moderate	moderate	Patient age should follow general trial criteria, and an upper age limitation of 85 years may be appropriate.
	No upper age limit	4	7	9	2	8.2	100%	Appr	high	moderate	
	Comment: if it meets other criteria	1									
4	**Stratifications**										
	Concurrent chemotherapy	4	2	9	7	6.5	75%	Appr	moderate	high	Panel disagrees. No concurrent chemotherapy allowed (see # 16).
	Other stratifications	1								high	Stratification required for induction chemotherapy vs. none (see also # 16).
	Comment: Induction or Adjuvant Chemotherapy - Stratification										

		Vote 1 and 2								Expert Panel	
	Criteria	Nr of Votes	Min Rank	Max Rank	Range of ranks	Mean Rank	%Agreement with Vote Category	Vote Category	Agreement	Exp. Panel Consensus	Exp. Panel Conclusions
5	**EXCLUSION Criteria: These tumor histologies and sites should be EXCLUDED from an SFRT trial**										
	Rhabdomyosarcoma	4	1	7	6	3.2	75%	Not appr	moderate	high	Eligible histologies should include undifferentiated pleomorphic sarcoma, myxoid liposarcoma and leiomyosarcoma. Extraskeletal osteo-sarcoma, which is rare, should be excluded<br><br>Eligible histologies should be consistent with those in the major prior sarcoma trials, which have excluded the histologies noted in #5.
	Ewing's sarcoma	4	3	9	6	6.5	75%	Appr	moderate		
	Comment:<br>Chemo sensitive	1									
	Chondrosarcoma	4	2	7	5	5.2	50%	Appr	low		
	Kaposi's sarcoma	4	3	9	6	5.5	50%	Appr<br>Not appr	low		
	Comment:<br>Only if bulky, these tend to be superficial skin	1									
	Angiosarcoma	4	2	9	7	6.0	50%	Appr	low		
	Comment:<br>If it meets other criteria	1									
	Malignant peripheral nerve sheath tumor	4	4	7	3	5.8	75%	May be appr	high		
	Comments:<br>1. If it meets other criteria<br>2. Depends on location; may be tricky in brachial plexus	2									

	Criteria	Vote 1 and 2								Expert Panel	
	Criteria	Nr of Votes	Min Rank	Max Rank	Range of ranks	Mean Rank	%Agreement with Vote Category	Vote Category	Agreement	Exp. Panel Consensus	Exp. Panel Conclusions
5	**EXCLUSION Criteria: These tumor histologies and sites should be EXCLUDED from an SFRT trial – con't**										
	Tumor site: Head & neck	4	1	8	7	4.5	50%	Appr<br>Not appr	low	high	Eligible primary tumor location should be consistent with major pilot trials.<br><br>Only extremity sarcomas should be eligible, to provide consistency of the study population and because the vast majority of clinical SFRT experience is in extremity sarcomas.
	Tumor site: Intraabdominal/retro-peritoneal	4	2	9	7	5.5	50%	Appr<br>Not appr	low		
	Comment:<br>Depends on proximity to kidneys and spinal cord	1									

	Criteria	Vote 1 and 2								Expert Panel	
	Criteria	Nr of Votes	Min Rank	Max Rank	Range of ranks	Mean Rank	%Agreement with Vote Category	Vote Category	Agreement	Exp. Panel Consensus	Exp. Panel Conclusions
6	**EXCLUSION Criteria: These conditions should be EXCLUDED from an SFRT trial**										
	Recurrent sarcoma (after prior radiation)	4	1	9	8	5.2	50%	Appr	low	high	Recurrent sarcoma after prior radiation and recurrent sarcoma after prior surgery should be excluded to reduce confounding variables in a clinical trial.
	Recurrent sarcoma (after prior surgery)	4	1	8	7	4.0	50%	Not appr	low		
	Prior chemotherapy	4	1	6	5	2.8	75%	Not appr	high	high	Agreed.
	Scleroderma (Systemic sclerosis)	4	3	8	5	6.0	50%	Appr	low	high	Patients with scleroderma should be excluded from a trial because of the increased toxicity risk, particularly to skin and subcutaneous regions.
	Comments:<br>1. If it meets other criteria<br>2. May not tolerate such high dose	2									

7	**Pre-treatment Evaluations: These investigations should be required**										
	CT (sarcoma site)	4	7	9	2	8.5	100%	Appr	high	high	Agreed.
	MRI (sarcoma site)	4	9	9	0	9.0	100%	Appr	high	high	Agreed.
	CT Chest/abdomen/pelvis	4	8	9	1	8.8	100%	Appr	high	high	Agreed.
	PET/CT (whole body)	4	7	9	2	7.8	100%	Appr	high	high	Agreed.

	Criteria	Vote 1 and 2								Expert Panel	
		Nr of Votes	Min Rank	Max Rank	Range of ranks	Mean Rank	%Agreement with Vote Category	Vote Category	Agreement	Exp. Panel Consensus	Exp. Panel Conclusions
8	**Radiation Therapy – SFRT: Dose**										
	15 Gy in 1 fraction	3	7	9	2	8.3	100%	Appr	high	high	A dose range of 15-18 Gy in 1 fraction is appropriate.
	18 Gy in 1 fraction	3	5	8	3	7.0	67%	Appr	high		
	15-18 Gy in 1 fraction	4	7	9	2	8.2	100%	Appr	high		
	Other SFRT dose:	1								high	EUD should be determined for SFRT according to the recent physics publications (Zhang et al, 2020, Xu et al. 2020). For computation the Modified Linear Quadratic Model is preferred. This may refine treatment in the future, as currently mechanisms of how SFRT may increase toxicity are still unknown.
	Comment: Often give 20 Gy in 1 fraction									high	The dose of 20 Gy in 1 fraction has been used but is more common in the palliative setting.

		Vote 1 and 2								Expert Panel	
	**Criteria**	**Nr of Votes**	**Min Rank**	**Max Rank**	**Range of ranks**	**Mean Rank**	**%Agreement with Vote Category**	**Vote Category**	**Agreement**	**Exp. Panel Consensus**	**Exp. Panel Conclusions**

9	**Radiation Therapy – SFRT: Target volume**										
	GTV (primary tumor) without margin	4	9	9	0	9.0	100%	Appr	high	high	Agreed.
	Additional boost (post-op) optional	4	1	9	8	4.0	75%	Not appr	moderate	high	Not appropriate for all cases, only for involved margins
	Comment:<br>Often give additional dose to 50 Gy to tumor per RTOG	1									
	Additional boost for involved margin only (per NRG 0630 guideline)	4	1	7	6	3.5	75%	Not appr	moderate	high	A post-operatively boost should only be permitted for involved margins
	Comment:<br>Depending on structures	1									

	Criteria	Vote 1 and 2								Expert Panel	
		Nr of Votes	Min Rank	Max Rank	Range of ranks	Mean Rank	%Agreement with Vote Category	Vote Category	Agreement	Exp. Panel Consensus	Exp. Panel Conclusions
10	**Radiation Therapy – SFRT: Normal OAR structures**										
	Exclude spinal cord	4	7	9	2	8.5	100%	Appr	high	high	Agreed.
	Exclude brachioplexus	4	3	6	3	4.8	75%	May be appr	high	high	Agreed.
	Comments:<br>1. Relative to tumor location<br>2. Can include if already or impending deficit/ paralysis	2									
	No treatment of the entire extremity circumference	4	1	9	8	4.0	75%	Not appr	moderate	high	The SFRT target volume must never include the entire skin circumference.
	Dose reduction to traumatized skin	4	1	9	8	5.5	50%	Appr	low	high	While skin doses are generally low for GRID fields because of the megavoltage beam's skin sparing effect, the skin surface dose from the SFRT should be <150% of the prescribed SFRT dose (maximally 30 Gy based on prior brachy-therapy experience).

		Vote 1 and 2								Expert Panel	
	**Criteria**	**Nr of Votes**	**Min Rank**	**Max Rank**	**Range of ranks**	**Mean Rank**	**%Agreement with Vote Category**	**Vote Category**	**Agreement**	**Exp. Panel Consensus**	**Exp. Panel Conclusions**
11	**Radiation Therapy – SFRT: SFRT technique**										
	GRID (collimator-based)	4	6	9	3	8.2	75%	Appr	high	high	Agreed.
	GRID (MLC-based)	4	9	9	0	9.0	100%	Appr	high	high	Agreed.
	GRID (either with same trial)	4	7	9	2	8.2	100%	Appr	high	high	Agreed.
	Comment: Stratify	1									Agreed (see #4)
	Lattice	4	3	7	4	5.2	50%	Appr	low	high	For an initial clinical trial, GRID therapy should be the technology of choice, because there is insufficient clinical experience with Lattice therapy in sarcoma.<br><br>Lattice therapy may be appropriate in future trials. Dose profiles are expected to show major differences with Lattice therapy. Valley dose may be higher than with GRID, and special care must be directed towards critical normal structures.
	Comment: Need more published clinical experience	1								high	Agreed.

		Vote 1 and 2								Expert Panel	
	**Criteria**	**Nr of Votes**	**Min Rank**	**Max Rank**	**Range of ranks**	**Mean Rank**	**%Agreement with Vote Category**	**Vote Category**	**Agreement**	**Exp. Panel Consensus**	**Exp. Panel Conclusions**
12	**Radiation Therapy – Conventional EBRT: Dose and technique**										
	PTV: 50 Gy in 25 fractions	4	7	9	2	8.5	100%	Appr	high	high	Agreed.
	Comment: Or 50.4 Gy in 28...commonly used in publications	1									
	IMRT	4	9	9	0	9.0	100%	Appr	high	high	Agreed.
	Comment: Per RTOG	1									
	IMRT or 3D Conformal	4	4	9	5	7.5	75%	Appr	high	high	Agreed.
	Comment: Per RTOG	1									
	No treatment of the entire extremity circumference	4	1	9	8	4.0	75%	Not appr	moderate	high	Agreed.

		Vote 1 and 2								Expert Panel	
	Criteria	Nr of Votes	Min Rank	Max Rank	Range of ranks	Mean Rank	%Agreement with Vote Category	Vote Category	Agreement	Exp. Panel Consensus	Exp. Panel Conclusions
13	**Radiation Therapy – Conventional EBRT: OAR constraints**										
	Conventional constraints without consideration for SFRT dose	4	1	9	8	6.8	75%	Appr	moderate	moderate	The dose contribution from the SFRT should not be counted towards the dose constraints. If there is concern regarding normal tissue doses, the SFRT to normal structures should be adjusted accordingly.

	Criteria	Vote 1 and 2								Expert Panel	
		Nr of Votes	Min Rank	Max Rank	Range of ranks	Mean Rank	%Agreement with Vote Category	Vote Category	Agreement	Exp. Panel Consensus	Exp. Panel Conclusions
14	**On-therapy Evaluations: Evaluate feasibility of correlative studies**										
	Toxicity assessment (weekly)	4	9	9	0	9.0	100%	Appr	high	high	Agreed.
	Correlative studies (blood, urine): pre-RT, 1 x during RT, post-tx	4	5	9	4	7.8	75%	Appr	high	high	Agreed.
	Correlative studies (blood, urine): pre-RT, 3 x during RT, post-RT	4	3	9	6	6.0	50%	Appr	low	high	While serial blood draws during the treatment course are not standard-of-care in radiation therapy for sarcoma, collection of blood and urine for correlative studies is acceptable and feasible.
	Tumor biopsy twice during radiation therapy	4	1	4	3	2.2	75%	Not appr	high	high	Agreed.
	Comment: Hard to do in practice	1									
	Normal tissue biopsy twice during radiation therapy	4	1	4	3	2.2	75%	Not appr	high	high	Agreed.
	Comment: Hard to do in practice	1									
	Quality of life assessment	4	8	9	1	8.8	100%	Appr	high	high	Agreed.
	Patient reported outcomes	4	6	9	3	8.2	75%	Appr	high	high	Agreed.

	Criteria	Vote 1 and 2 Nr of Votes	Min Rank	Max Rank	Range of ranks	Mean Rank	%Agreement with Vote Category	Vote Category	Agreement	Expert Panel Exp. Panel Consensus	Exp. Panel Conclusions
15		Systemic Therapy: Agents									
	Ifosfamide	4	4	9	5	7.2	75%	Appr	high	high	Any neoadjuvant and adjuvant chemotherapy agent considered acceptable in standard-of-care practice is allowed
	Comment: Need to base on published data for all chemoRx listed	1									
	Doxorubicin	3	4	9	5	6.3	67%	May be appr	high		
	Comment: Have to watch for skin toxicity (recall)	1									
	Gemcitabine	2	4	9	5	6.5	50%	Appr<br>May be appr	low		
	Dacarbazine	3	4	9	5	7.0	67%	Appr	high		
	Cyclophosphamide	3	4	9	5	7.0	67%	Appr	high		
	Doxorubicin/ Ifosfamide/ Mesna (AIM)	3	4	9	5	7.0	67%	Appr	high		
	Comment: Watch for skin toxicity	1									
	Doxorubicin/ Ifosfamide/ Dacarbazine/ Mesna (MAID)	3	4	9	5	7.0	67%	Appr	high		
	Comment: Watch for skin toxicity with adriamycin	1									
	Gemcitabine/ Docetaxel	3	4	9	5	6.7	67%	Appr	high		
	Gemcitabine/ Vinorelbine	3	4	9	5	6.7	67%	Appr	high		
	Gemcitabine/ Dacarbazine	3	4	9	5	6.7	67%	Appr	high		
	Other systemic therapy agents	1									
	Comment: Chemo per medical oncologist discretion										

		Vote 1 and 2								Expert Panel	
	**Criteria**	**Nr of Votes**	**Min Rank**	**Max Rank**	**Range of ranks**	**Mean Rank**	**%Agreement with Vote Category**	**Vote Category**	**Agreement**	**Exp. Panel Consensus**	**Exp. Panel Conclusions**
16	**Systemic Therapy: Timing**										
	Chemotherapy during radiation therapy permitted	4	1	7	6	4.8	50%	Appr	low	high	Concurrent chemotherapy (during radiation therapy) is not permitted in order to reduce confounding variables in an initial clinical trial.<br>Concurrent chemotherapy has not been used in in 2 of the 3 pilot studies, and is inconsistently and not widely used in standard-of-care practice. Thus the exclusion of concurrent chemotherapy should also not significantly impair trial accrual.<br>Concurrent chemotherapy can be considered in a subsequent trial, once results from initial trials are available.
	Comment:<br>Little published data	1									
	Chemotherapy DURING THE SFRT FRACTION permitted	4	1	3	2	1.5	100%	Not appr	high	high	Concurrent chemotherapy is not permitted
	Adjuvant chemotherapy permitted	4	7	9	2	8.2	100%	Appr	high	high	Agreed.
	Neoadjuvant systemic therapy NOT permitted	4	1	9	8	5.0	50%	May be appr	low	high	Panel disagrees:<br>Neoadjuvant chemo-therapy is permitted.

		Vote 1 and 2								Expert Panel	
	**Criteria**	**Nr of Votes**	**Min Rank**	**Max Rank**	**Range of ranks**	**Mean Rank**	**%Agreement with Vote Category**	**Vote Category**	**Agreement**	**Exp. Panel Consensus**	**Exp. Panel Conclusions**
**17**	**Systemic Therapy: Immunotherapy**										
	Immunotherapy as part of trial regimen	4	3	8	5	5.2	50%	May be appr	low	high	Immunotherapy is not permitted in an initial in order to reduce variables that may confound endpoints in an initial clinical trial.<br><br>Immunotherapy should be studied in a subsequent trial.
	Comments:<br>1. Another trial<br>2. Would wait for after a pure trial done	2									

	Criteria	Vote 1 and 2								Expert Panel	
		Nr of Votes	Min Rank	Max Rank	Range of ranks	Mean Rank	%Agreement with Vote Category	Vote Category	Agreement	Exp. Panel Consensus	Exp. Panel Conclusions
**18**	**Post-radiation Therapy (preoperative) Evaluations: Response assessment**										
	Clinical exam	4	8	9	1	8.8	100%	Appr	high	high	Agreed.
	Imaging response (MRI): Tumor size (RECIST)	4	8	9	1	8.8	100%	Appr	high	high	Agreed.
	Imaging response (MRI): >90% tumor necrosis	4	5	9	4	7.8	75%	Appr	high	high	Agreed.
	Timing: May be appr weeks after radiation	4	8	9	1	8.8	100%	Appr	high	high	Agreed.
	QOL assessment	4	8	9	1	8.8	100%	Appr	high	high	Agreed.
	Patient reported outcomes	4	9	9	0	9.0	100%	Appr	high	high	Agreed.

**19**	**Surgical Evaluation: Pathologic response**										
	Resectability	4	9	9	0	9.0	100%	Appr	high	high	Agreed.
	R0 vs R1 resection	4	9	9	0	9.0	100%	Appr	high	high	Agreed.
	Other Evaluation (path response)	2								high	Pathologic response should be evaluated by quantitative histologic assessment of percentage of necrosis >90-95%), as used in standard-of-care practice.
	Comments:<br>1. Pathologic necrosis rate<br>2. Necrosis rate seen pathologically										

	Criteria	Vote 1 and 2								Expert Panel	
		Nr of Votes	Min Rank	Max Rank	Range of ranks	Mean Rank	%Agreement with Vote Category	Vote Category	Agreement	Exp. Panel Consensus	Exp. Panel Conclusions

20	Post-(all)therapy Evaluations: Clinical										
	Clinical exam	4	8	9	1	8.8	100%	Appr	high	high	Agreed.
	Toxicity assessment	4	9	9	0	9.0	100%	Appr	high	high	Agreed.
	Every 3 months (year 1-2)	4	8	9	1	8.8	100%	Appr	high	high	Agreed.
	Every May be appr months (year 3-5)	4	8	9	1	8.8	100%	Appr	high	high	Agreed.
	QOL assessment	4	9	9	0	9.0	100%	Appr	high	high	Agreed.
	Patient reported outcomes	4	9	9	0	9.0	100%	Appr	high	high	Agreed.

21	Post-(all)therapy Evaluations: Imaging										
	CT (site-specific)	4	5	9	4	7.0	50%	Appr<br>May be appr	low	high	Imaging response should be evaluated by standard-of-care modalities, preferably MRI, which is in wide use.
	Comment:<br>chest	1									
	MRI (site-specific)	4	8	9	1	8.8	100%	Appr	high	high	Agreed.
	Comment:<br>Primary tumor	1									Agreed.
	CT chest/ abdomen/pelvis	4	7	9	2	8.2	100%	Appr	high	high	Agreed.
	Comment:<br>Retroperitoneal sarcoma	1									N/A – only extremity sarcoma eligible
	Imaging every 4 months	4	5	9	4	6.8	50%	Appr<br>May be appr	low	high	Every 3-4 mo. for 2 years; every 6 mo, for 3 years, then annually.

22	Knowledge gaps in SFRT for sarcoma	
	Clinical:	Experience in actual clinical use
		Effectiveness in increasing pathologic CR rates
		Potential positive local and systemic effects and local long term toxicity
	Physics:	Appropriate field set up and treatment delivery
		Delivery and QA
	Biology:	Radiobiologic effect

23	Demographics of Voters

I am practicing or have practiced clinical SFRT in patient care.
I am practicing or have practiced clinical SFRT in sarcoma patients.
I have presented abstract(s) on clinical SFRT including patient outcomes.
I have presented abstract(s) on clinical SFRT including patient outcomes in sarcoma patients.
I have published scientific article(s) on clinical SFRT including patient outcomes.
I have published scientific article(s) on clinical SFRT including patient outcomes in sarcoma patients.
Other: Preparing publications

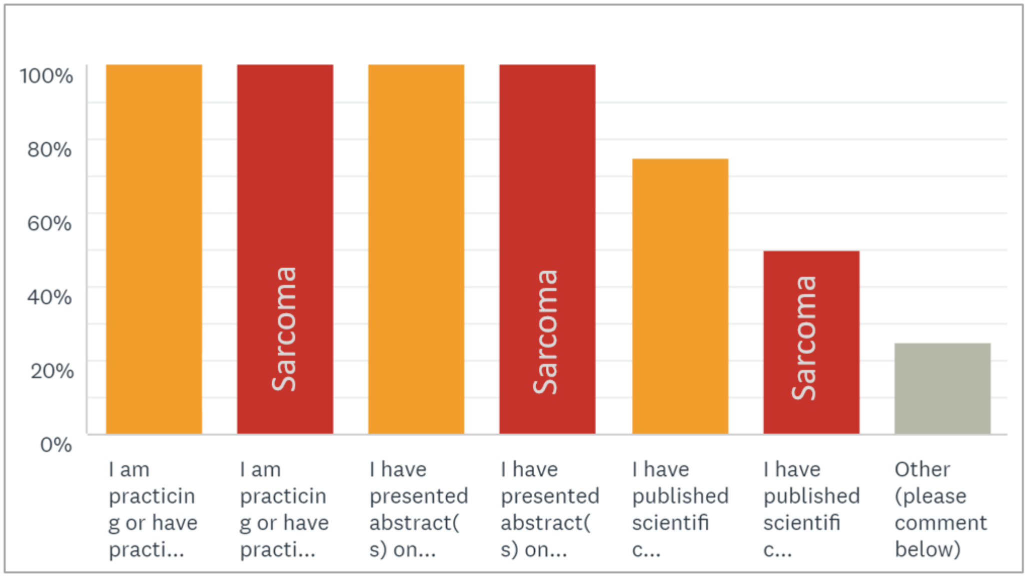

Supplement: Supplementary file 4 [file mmc4.pdf]
