# Supplementary material for: An International Consensus on the Design of Prospective Clinical–Translational Trials in Spatially Fractionated Radiation Therapy
Source: Adv Radiat Oncol. 2021 Dec 11;7(2):100866. doi: 10.1016/j.adro.2021.100866 (PMC8843999; doi:10.1016/j.adro.2021.100866)
Supplement: Supplementary file 5 [file mmc5.docx]

**Appendix 5:**

**Consensus Guideline Recommendations for the Design of Clinical Trials in**

**Spatially Fractionated Radiation Therapy for Head and Neck Cancer**

**Introduction**

Spatially fractionated radiation therapy (SFRT), the treatment of tumors with intentionally non-uniform dose, is a complex radiotherapy concept of increasing interest in clinical and experimental radiation oncology. Pilot studies show high tumor response and low toxicity with SFRT in patients treated with palliative or curative intent for bulky tumors ^1-6^ including head and neck (H&N) cancer ^1-3^. High local/regional control rates in the neck of 79-92%, and survival rates of 50-79% were seen in these studies ^4 5^. These outcomes in very advanced tumors compare favorably with the regional control rates of 25-66% and survival rates as low as of 30% reported with conventional radiation and radiation or radio-chemotherapy ^7 8^, and particularly with the continued poor outcomes in patients with matted lymph node involvement and bilateral neck disease ^9-11^. However, no prospective randomized or multi-institutional clinical trials of SFRT have been conducted. Consensus on complex SFRT clinical trial design parameters is essential to enable broad participation and successful accrual in future SFRT trials, while facilitating trial designs that incorporate relevant physics metrics as well as enable translational studies of SFRT. Such consensus is challenged by the highly variable SFRT technologies and techniques, the complex dosing concepts, and the overall still limited clinical experience with SFRT in the definitive treatment of specific primary malignancies. The purpose of this guideline was to develop a common approach for future multi-institutional clinical trial design in SFRT specific to H&N cancer.

Following an initial literature review, the consensus was developed by a group of recognized SFRT experts who rated a comprehensive set of clinical trial design categories (detailed in the guideline) through anonymous electronic voting. Voting results were shared among a H&N cancer specific Expert Panel, consisting of 3 radiation oncologists, one physicist and one biologist. The voting results were iteratively reviewed and discussed by the Expert Panel, followed a second voting round, re-discussion of the voting results, public comment posting, review and discussion of the comments, a repeat literature review, and the development of the final guideline recommendations presented here.

**SFRT Clinical Trial Design Consensus Guideline for H&N Cancer**

These clinical trial design recommendations are guided by three SFRT outcome studies of multiple disease sites containing head and neck (H&N) cancer patients ^1-3^, three *disease-specific* studies of H&N cancer patient cohorts ^4-6^, review of the overall SFRT literature as well as the clinician, physicist and biologist experience of the multidisciplinary Expert Panel for SFRT clinical trials in H&N cancer.

*Eligible Disease Sites*

Based on the patient characteristics of the published outcome studies ^4 5^, the Panel considers oropharynx and hypopharynx tumors appropriate (high consensus) for inclusion into clinical SFRT trials. Nasopharyngeal tumors are appropriate ^4 5^ (moderate consensus) and advanced skin primaries with bulky lymph node involvement can be included (high consensus). Uncommon primary sites, such as salivary gland and paranasal sinus tumors, should be excluded because of their different spread pattern, often variable histology and overall low incidence (high consensus). While oro-, hypo-, nasopharynx, supraglottic and glottic larynx primaries are considered eligible, it is recognized that there is currently insufficient clinical evidence in favor of specific individual H&N primary sites for inclusion into SFRT trials (high consensus).

*Eligibility/Exclusion criteria: Disease Stage, Tumor Size/Extent/invasion*

There was high consensus that eligible tumor stage/size is guided by the lymph node status, *not* the status (T-stage) of the primary site disease site. The Panel emphasizes that the vast majority of reported clinical experience ^1-6^ clinical practice with SFRT in H&N cancer is in the treatment of bulky lymph nodes (not the primary tumor). Patients with any T-stage *and* N3 lymph node stage, i.e. lymph node size of more than 6 cm, of either individually or matted lymph nodes, are eligible for an SFRT trial.

The Expert Panel strongly and unanimously recommends that tumors with both carotid invasion and skin involvement, or both carotid invasion and prior radiation therapy be excluded from clinical trials. This exclusion is based on the experience of fatal carotid bleeding in a patient with carotid invasion and prior radiation ^5^ and unpublished experience of fatal carotid bleeding in a patient with both carotid invasion and skin involvement after SFRT.

*Eligibility/Exclusion criteria: Histology*

Eligible histologies should include squamous cell carcinoma, based on the majority of the published clinical experience. Inclusion of patients with bulky tumors that are HPV (P16) positive should be considered (moderate consensus). Uncommon and highly radiosensitive histologies, such as sarcoma or lymphoma should be excluded (high consensus).

*Eligibility/Exclusion criteria: Prior treatment*

*Recurrent tumors after prior surgery*. Recurrent tumors after prior treatment may be included if the recurrence consists in bulky neck recurrence, and if the target region was not previously irradiated.

*Recurrent tumors after prior radiation therapy.* Consensus was moderate regarding prior radiation therapy. Exclusion of patients with prior radiation therapy was favored by the Panel (moderate consensus) to minimize potential confounding variables for of the outcome analysis. A separate subsequent clinical trial was suggested for patient populations with recurrence after prior radiation, to follow an initial trial with radiotherapy-naïve patients.

*Prior chemotherapy.* Prior chemotherapy, including neoadjuvant (induction) chemotherapy, is not recommended for clinical trial enrollment because of potential of variable responses prior to the trial regimen that may confound interpretation of the outcome endpoints.

*Eligibility/Exclusion criteria: Patient factors (age, toxicity risk factors)*

Enrollment of patients at least 18 years of age was agreed with high consensus, and no upper age limit, as long as performance status is acceptable, was favored. Individual patient risk factors for toxicity should be considered. Patients with scleroderma (systemic sclerosis) should be excluded (high consensus).

*Stratifications*

Recommended stratifications include T-stage, preferably stratified by grouping of stages T1/T2 vs. T3/T4 and HPV status. In this special case of a highly technology-dependent trial, stratification was recommended according to SFRT technology of GRID vs. Lattice therapy (if Lattice were to be used in the future) and individual GRID techniques (high consensus). No other disease or treatment parameters, such as concurrent chemotherapy, which is employed commonly in H&N cancer, were recommended for stratification.

*Endpoints*

Local control and toxicity are recommended as primary endpoints. The feasibility of delivering SFRT according to the dosimetric and physics specifications ^12^ (see section *Radiation Therapy: SFRT dose*), disease-specific survival, overall survival and quality of life outcome metrics can be considered as additional endpoints in an initial or subsequent trial.

*Pre-treatment Evaluations (clinical, imaging, histologic investigations)*

Pretreatment evaluation according to standard of care was recommended, including for imaging maxillofacial/neck CT and/or MRI and PET/CT (high consensus); swallowing study and fiber optic laryngoscopy where applicable; pertinent laboratory studies; and chest CT with the inclusion of upper abdomen/liver for metastatic workup. All enrolled patients should have HPV testing of their tumor (high consensus).

*Radiation Therapy – SFRT: Dose*

Based upon outcome data ^1-6^ the preferred SFRT schedule is a dose of 15 Gy in one fraction to the gross tumor target of the bulky lymph node(s). In two of the three published H&N cancer cohorts, 15 Gy was the most commonly used dose schedule and was associated with high local tumor control and a low level of toxicity ^4 6^, thus providing the basis for this recommendation. While a schedule of 20 Gy in 1 fraction has been used in one of the three disease-specific outcome studies in H&N cancer cohorts ^5^, and in a small proportion of patients in other studies ^3 4 6^, the higher dose of 20 Gy has been overall employed more commonly in the palliative setting ^1-3^. Therefore and because no dose response relationship favoring the higher dose is identifiable, the Panel considers 15 Gy in 1 fraction the preferred dosing regimen for an initial trial of definitive SFRT in H&N cancer (high consensus). While peripheral doses have not been routinely tracked in the published GRID therapy studies, they generally are in the range of 3 Gy for a 15 Gy SFRT dose ^13^. For prospective trials, tracking of the dose at the periphery of the GTV is recommended.

It is emphasized that the EUD must be determined for any trial dose regimen, particularly in view of different GRID technologies (collimator based and MLC-based), which have different dose distributions. While the role of EUD has not been explored in SFRT, the very principle of EUD is to assess non-uniform doses with respect to tissue effects. Therefore EUD is the most plausible and appropriate model for correlations with tumor control and normal tissue complication probability**.** MLC based GRID therapy may form a lattice-like GRID pattern if more than one gantry angle are used. EUDs can provide comparisons between plans and must be calculated for both tumor and normal tissues. While multiple methods are available to compute EUD, the modified linear quadratic model is favored, as further detailed in the respective SFRT physics guideline publications ^12 14^.

*Radiation Therapy – SFRT: Target volume*

The Panel recommends unanimously that the tumor target should consist in the bulky nodal mass, not in the primary tumor because of very scant outcome data of applying SFRT to the primary tumor. Based on the available clinical outcome data ^3-6^, the target (PTV) should include the GTV, consisting of the gross tumor of the lymph node mass by imaging, without an additional margin (high consensus).

*Radiation Therapy – SFRT: Normal Organ-at-Risk structures*

Based on published data ^3-6^ and the Panel’s clinical experience, critical normal organ-at-risk (OAR) structures, include spinal cord, brainstem and optic chiasm structures (high consensus). Consideration of brachial plexus, carotid artery and mandible as OARs may be appropriate (moderate consensus). Regarding the carotid artery, ineligibility of patients who have carotid involvement *and* skin involvement and/or carotid involvement *and* have received prior irradiation, should be noted (see section *Eligibility/Exclusion criteria: Disease stage, Tumor size/Extent/invasion*). The addition of PRV margins to the OAR structures can be considered, particularly to the spinal cord and brainstem (moderate consensus).

*Radiation Therapy – SFRT: Technique*

Both GRID technologies, collimator-based and MLC-based GRID therapy are the preferred SFRT technologies at this time. Collimator-based and MLC-based GRID may be applied within the same trial, under the condition that EUD has been determined and is comparable. While there was overall support for Lattice therapy as an SFRT technology in H&N cancer in the future, to date (at the time of this writing), there is no published data on the use of Lattice therapy in H&N cancer. While such published experience is expected to emerge, at this time the Panel favors GRID therapy technologies for an initial clinical trial.

*Radiation Therapy – Conventional ERT: Dose and technique*

Conventionally fractionated external beam radiation therapy (ERT) must be given immediately following SFRT, and it has been demonstrated that tumor response is inferior when SFRT is given without the addition of conventional radiation therapy ^1 2^. The conventional ERT should start within 72 hours of the SFRT fraction.

For the conventional radiotherapy portion of treatment, conventional definitive dose regimens, specific to the H&N disease site are applied as the dose prescription. PTV doses are generally in the range of 70-72 Gy to the primary gross tumor, 60-63 Gy to the high-risk subclinical target, and 50-56 Gy to the low-risk subclinical target (high consensus). In the SFRT literature for H&N cancer, the conventional doses to the gross tumor ranged from 66 Gy (combined with SFRT of 20 Gy/1 faction) ^5^; to 70 Gy (median; range 68‐79 Gy) ^4^; and 69.96 to 72.08 Gy in 2.12 Gy/fraction ^6^, with conventional doses to intermediate and low-risk PTVs ^5^. Reduction of the definitive conventional radiotherapy dose below standard dosing regimens is not recommended. In one study response rate was only 25% if conventional ERT doses were lower than 75% of the planned definitive dose ^6^.

The use of IMRT is encouraged (high consensus) and the use of a simultaneously integrated boost (SIB) was considered appropriate for other bulky areas of involvement, while cautioning that an SIB may add additional variability to the treatment regimen. If an SIB is used the dose to the SFRT GTV should be limited to 69.6 Gy in 33 fractions.

*Radiation Therapy – Conventional ERT: OAR constraints*

Dose constraints to OARs for the conventional ERT portion of treatment were recommended to follow those in standard practice without consideration of the dose contribution from the SFRT component of treatment (high consensus). SFRT contributions to OARs must be addressed during the planning of the SFRT component of treatment (see section *SFRT:* *Normal Organ-at-Risk structures*).

*On-therapy Evaluations: Evaluate feasibility*

On-treatment evaluations should include regular (customarily weekly) toxicity assessments, quality of life assessments and patient reported outcomes, along with routine imaging that typically includes CBCT imaging for response assessment and adaptive therapy as needed. CBCT imaging during treatment can be incorporated into trials to establish criteria for intra-treatment response assessment and adaptive therapy that may be required in SFRT ^15^.

Specimen collection of blood and urine at multiple times during radiation therapy for translational correlative science studies of SFRT should be strongly considered (high consensus). The collection of such specimens is feasible in a trial, particularly as patients, who commonly have concurrent chemotherapy, already undergo regular blood collections as the clinical standard of care, and this “liquid biopsy” concept can be leveraged for correlative science studies. While pre-therapy tumor biopsies are available for correlative studies, tumor tissue sampling *during* the treatment course was considered not to be clinically practical or generally feasible based on the potential clinical risk. If possible and available, advanced functional and molecular imaging techniques, such as vascular and metabolic imaging, may provide non-invasive and non-tissue-altering methods to characterize changes in functional tissue properties within the tumor volume in response to SFRT during ongoing radiation therapy, and for response evaluation after treatment ^16-19^.

*Systemic Therapy: Agents and timing*

Chemotherapy and targeted systemic therapy agents that are typically considered appropriate in conjunction with standard-fractionation radiation therapy for H&N cancer are acceptable for a clinical trial (high consensus). These agents typically include but may not be limited to platinum-based chemotherapies, Taxanes and Cetuximab. Chemotherapy can be given concurrently during the radiation therapy course for the conventionally fractionated component of radiation therapy. However, systemic therapy should *not* be given *during* the SFRT component of treatment (high consensus). Typical schedules that have been clinically employed consist of the SFRT fraction given first (without systemic therapy), followed by conventional radiation therapy/concurrent systemic therapy start within 72 hours. This can be accomplished, for example by delivering the SFRT fraction on a Friday and starting concurrent radiation/systemic therapy on the following Monday.

*Systemic Therapy: Immunotherapy*

There is no published experience with the combination SFRT and immunotherapy. There has been no consensus among the voters on combinations of SFRT and immunotherapy, and the Panel favors not include immunotherapy for an initial trial (moderate consensus). Combinations with immunotherapy, which are of particular interest in SFRT from a biology standpoint, should be tested in a subsequent trial, and be guided by the ablative stereotactic radiation and immunotherapy experience and future SFRT/immunotherapy experience.

*Post-therapy Evaluations: Clinical, imaging*

Overall, post-therapy response and outcome assessments follow generally accepted clinical standards. Clinical evaluation includes pertinent physical examination that may include fiber-optic exams as indicated for response and toxicity assessments (high consensus).

The outcome endpoint of local/regional control in the neck is important but can be challenging to definitively characterize because of inter-institutional variability in response assessment and the use and timing of post-radiation neck dissection. Determination of local control should be based on the 3-month post-therapy PET/CT (using established response criteria), and the need for post-radiation neck dissection, including pathologic response at the time of neck dissection.

Trial assessments for quality-of-life and patient reported outcomes are recommended (high consensus). Imaging studies maxilla/facial/neck CT and/or MRI and a 3-month post therapy PET/CT was recommended.

**Knowledge Gaps that May be Addressed through SFRT Clinical Trials in H&N Cancer**

*Clinical* knowledge gaps identified by consensus voters and Expert Panel include a better understanding of SFRT dose and fractionation; tolerance to SFRT; and appropriate combination therapies and optimal inclusion of chemotherapy/immunotherapy into SFRT regimens. The differences and impact of SFRT on systemic and local control outcomes is not sufficiently understood and is well suited to be addressed by prospective clinical trials.

As current clinical data are primarily based on SFRT for the treatment of bulky lymph nodes, the potential role of SFRT for the treatment of the primary tumor remains an open question, as is the role of SFRT in patients with moderate bulk of disease. Physician education is an unmet need.

Knowledge gaps in the *physics* of SFRT focus on standardization of SFRT delivery systems, and the standardization of SFRT’s unique dosimetric metrics (as detailed in recent guidelines ^12^) to be applied in clinical trials. The use of *standardized* metrics in clinical trials is critical to allow robust correlations of heterogeneous dose properties with tumor and normal tissue outcomes, to broaden our understanding of heterogeneous dose properties and response, and to develop optimized dose prescription.

Knowledge gaps in area of *biology* include mechanisms of action; immunological effects; the elucidation of biological cues that can be harnessed for improved outcomes; and the exploration of tumor and normal tissue volume effects on response.

**Conclusion**

SFRT clinical trials in H&N cancer are feasible based on the clinical experience provided by the pilot studies. Recommendations for eligibility aim to establish a uniform patient cohort of advanced oropharynx, larynx and nasopharynx primaries with bulky lymph node involvement, while excluding uncommon primary sites and histologies to minimize confounding variables that may hamper the interpretation of the outcome results. Patients with squamous cell carcinoma, both HPV-negative and HPV-positive, should be enrolled. The current experience supports SFRT to bulky *lymph nodes* rather than the primary tumor. GRID technology is favored over Lattice radiotherapy based the technologies used in current pilot studies. A single SFRT fraction of 15 Gy is recommended, and is followed by full-dose conventional (uniform) external beam radiation therapy. Reporting of inhomogeneity dose parameters according to recent SFRT physics guidelines, particularly EUD is highly recommended to allow data interpretation, plan comparison and correlation of dose parameters with clinical outcome. Concurrent chemotherapy agents used in standard-of-care are permitted for the conventional (uniform) external beam radiation therapy of treatment, not for the SFRT component. Pre-therapy, on-therapy and post-therapy investigations to assess tumor control and toxicity endpoints generally follow the standard of care, and should include patient reported outcomes. Specimen collection (blood, urine), synchronized prospectively with the treatment course, for translational correlative science studies is highly recommended. However aside from pre-therapy (diagnostic) biopsies and post-therapy tissue in cases of recurrent disease, tumor tissue collection during therapy for correlative science is challenging in the current clinical environment.

**References**

1. Mohiuddin M, Fujita M, Regine WF, et al. High-dose spatially-fractionated radiation (GRID): a new paradigm in the management of advanced cancers. *Int J Radiat Oncol Biol Phys* 1999;45(3):721-7. doi: 10.1016/s0360-3016(99)00170-4

2. Mohiuddin M, Stevens JH, Reiff JE, et al. Spatially fractionated (GRID) radiation for palliative treatment of advanced cancer. *Radiation Oncocogy Investigations* 1996;4:41-47.

3. Neuner G, Mohiuddin MM, Vander Walde N, et al. High-dose spatially fractionated GRID radiation therapy (SFGRT): a comparison of treatment outcomes with Cerrobend vs. MLC SFGRT. *Int J Radiat Oncol Biol Phys* 2012;82(5):1642-9. doi: 10.1016/j.ijrobp.2011.01.065

4. Huhn JL, Regine WF, Valentino JP, et al. Spatially fractionated GRID radiation treatment of advanced neck disease associated with head and neck cancer. *Technol Cancer Res Treat* 2006;5(6):607-12. doi: 10.1177/153303460600500608

5. Penagaricano JA, Moros EG, Ratanatharathorn V, et al. Evaluation of spatially fractionated radiotherapy (GRID) and definitive chemoradiotherapy with curative intent for locally advanced squamous cell carcinoma of the head and neck: initial response rates and toxicity. *Int J Radiat Oncol Biol Phys* 2010;76(5):1369-75. doi: 10.1016/j.ijrobp.2009.03.030

6. Choi JI, Daniels J, Cohen D, et al. Clinical Outcomes of Spatially Fractionated GRID Radiotherapy in the Treatment of Bulky Tumors of the Head and Neck. *Cureus* 2019;11(5):e4637. doi: 10.7759/cureus.4637

7. Mendenhall WM, Million RR, Cassisi NJ. Squamous cell carcinoma of the head and neck treated with radiation therapy: the role of neck dissection for clinically positive neck nodes. *Int J Radiat Oncol Biol Phys* 1986;12(5):733-40. doi: 10.1016/0360-3016(86)90030-1 [published Online First: 1986/05/01]

8. Witek ME, Wieland AM, Chen S, et al. Outcomes for patients with head and neck squamous cell carcinoma presenting with N3 nodal disease. *Cancers Head Neck* 2017;2 doi: 10.1186/s41199-017-0027-z

9. Goguen LA, Posner MR, Tishler RB, et al. Examining the need for neck dissection in the era of chemoradiation therapy for advanced head and neck cancer. *Arch Otolaryngol Head Neck Surg* 2006;132(5):526-31. doi: 10.1001/archotol.132.5.526

10. Spector ME, Chinn SB, Bellile E, et al. Matted nodes as a predictor of distant metastasis in advanced-stage III/IV oropharyngeal squamous cell carcinoma. *Head Neck* 2016;38(2):184-90. doi: 10.1002/hed.23882

11. Vainshtein JM, Spector ME, Ibrahim M, et al. Matted nodes: High distant-metastasis risk and a potential indication for intensification of systemic therapy in human papillomavirus-related oropharyngeal cancer. *Head Neck* 2016;38 Suppl 1:E805-14. doi: 10.1002/hed.24105

12. Zhang H, Wu X, Zhang X, et al. Photon GRID Radiation Therapy: A Physics and Dosimetry White Paper from the Radiosurgery Society (RSS) GRID-Lattice-Microbeam-FLASH Radiotherapy Working Group. *Radiat Res* 2020 doi: 10.1667/RADE-20-00047.1

13. Zhang H, Wang JZ, Mayr N, et al. Fractionated grid therapy in treating cervical cancers: conventional fractionation or hypofractionation? *Int J Radiat Oncol Biol Phys* 2008;70(1):280-8. doi: 10.1016/j.ijrobp.2007.08.024

14. Wu X, Perez N, Zheng Y, et al. The Technical and Clinical Implementation of LATTICE Radiation Therapy (LRT). *Radiat Res* 2020 doi: 10.1667/RADE-20-00066.1

15. Amendola BE, Perez NC, Mayr NA, et al. Spatially Fractionated Radiation Therapy Using Lattice Radiation in Far-advanced Bulky Cervical Cancer: A Clinical and Molecular Imaging and Outcome Study. *Radiat Res* 2020 doi: 10.1667/RADE-20-00038.1

16. Cooper RA, Carrington BM, Loncaster JA, et al. Tumour oxygenation levels correlate with dynamic contrast-enhanced magnetic resonance imaging parameters in carcinoma of the cervix. *Radiother Oncol* 2000;57(1):53-9. doi: 10.1016/s0167-8140(00)00259-0

17. Egeland TA, Simonsen TG, Gaustad JV, et al. Dynamic contrast-enhanced magnetic resonance imaging of tumors: preclinical validation of parametric images. *Radiat Res* 2009;172(3):339-47. doi: 10.1667/RR1787.1

18. Padhani AR. Dynamic contrast-enhanced MRI in clinical oncology: current status and future directions. *J Magn Reson Imaging* 2002;16(4):407-22. doi: 10.1002/jmri.10176

19. Wang P, Popovtzer A, Eisbruch A, et al. An approach to identify, from DCE MRI, significant subvolumes of tumors related to outcomes in advanced head-and-neck cancer. *Med Phys* 2012;39(8):5277-85. doi: 10.1118/1.4737022
