# Supplementary material for: An International Consensus on the Design of Prospective Clinical–Translational Trials in Spatially Fractionated Radiation Therapy
Source: Adv Radiat Oncol. 2021 Dec 11;7(2):100866. doi: 10.1016/j.adro.2021.100866 (PMC8843999; doi:10.1016/j.adro.2021.100866)
Supplement: Supplementary file 6 [file mmc6.docx]

**Appendix 6:**

**Consensus Guideline Recommendations for the Design of Clinical Trials in**

**Spatially Fractionated Radiation Therapy for Soft Tissue Sarcoma**

**Introduction**

Spatially fractionated radiation therapy (SFRT), the treatment of tumors with intentionally non-uniform dose, is a complex radiotherapy concept of increasing interest in clinical and experimental radiation oncology. Pilot studies show high tumor response and low toxicity with SFRT in patients treated with palliative or curative intent for bulky tumors, including sarcoma ^1-9^. These studies show high response rates, local control rates of 85-100% ^4 6^ and a limb-sparing rate of 93% ^4^ in bulky (>8-10 cm) sarcomas. These results exceed outcomes with current standard therapy in patients in bulky (>8 cm) soft tissue sarcomas ^10-12^. In contrast, with conventional preoperative radiation of 50 Gy, only 10% of patients with bulky sarcomas (>10 cm) attain local control at 5 years ^13^, and in high-grade tumors the median pathologic tumor necrosis is only 50% ^14^, well below the necrosis rate of >90% that is widely recognized as predictor of tumor control and survival ^10 15^. Conventional dose escalation to >63Gy ^13^ or intensification of chemotherapy ^16^ is desirable but is associated with higher early and long term toxicity and therefore not routinely performed. While the SFRT pilot data suggest improvement, no prospective randomized or multi-institutional clinical trials of SFRT have been conducted in bulky soft tissue sarcoma. Consensus on complex SFRT clinical trial design parameters is essential to enable broad participation and successful accrual in future SFRT trials, while facilitating trial designs that incorporate relevant physics metrics as well as enable translational studies of SFRT. Such consensus is challenged by the highly variable SFRT technologies and techniques, the complex dosing concepts, and the overall still limited clinical experience with SFRT in the definitive treatment of specific *primary* malignancies. The purpose of this guideline was to develop a common approach for future multi-institutional clinical trial design in SFRT specific to soft tissue sarcoma.

Following an initial literature review, the consensus was developed by a group of recognized SFRT experts who rated a comprehensive set of clinical trial design categories (detailed in the guideline) through anonymous electronic voting. Voting results were shared among a Sarcoma-specific Expert Panel, consisting of 3 radiation oncologists, one physicist and one biologist. The voting results were iteratively reviewed and discussed by the Expert Panel, followed by public comment posting, review and discussion of the comments, a repeat literature collection and review, and the development of the final guideline recommendations presented here.

**SFRT Clinical Trial Design Consensus Guideline for Soft Tissue Sarcoma**

The SFRT clinical trial design recommendations are guided by studies of multiple disease sites containing sarcoma patients and by disease-specific studies of cohorts that include only sarcoma patients. The clinical experience to date consists of two published studies with cohorts of largely palliatively treated patients that contain patients with soft tissue sarcoma ^1 2^. *Disease-specific* series of definitively treated patients with sarcoma have been presented in abstract form ^4 5^, and one outcome study ^6^ was recently published. Collectively these clinical cohorts provide consistent pilot experience that was considered in conjunction with the clinician, physicist and biologist experience of the multidisciplinary Expert Panel for SFRT Clinical Trials in Sarcoma.

*Eligible disease sites*

A clinical trial of SFRT and sarcoma should include predominantly soft-tissue sarcomas of the extremities, the most common presentation, which also have the most pilot experience. Inclusion of the less common head and neck region and intraabdominal/retroperitoneal disease sites was considered to add unnecessary variability for the interpretation of outcomes in a clinical trial (high consensus).

*Eligibility/Exclusion criteria: Disease stage, Tumor size/Extent/invasion*

Enrollment of patients with unresectable grade 2-3 soft tissue sarcomas, stages IB–IIIB, bulky >8 cm of the extremity (high consensus), who are planned for a treatment regimen including pre-operative radiation, is recommended (high consensus). It may be appropriate to enroll patients with lymph node involvement, which is overall uncommon (­­high consensus).

*Eligibility/Exclusion criteria: Histology*

Patients with undifferentiated pleomorphic sarcoma, myxoid liposarcoma and leiomyosarcoma should be considered eligible (high consensus). This eligibility profile reflects that of major randomized prior trials in sarcoma with conventional radiation therapy ^11 12 17^. The Expert Panel considered it important to maintain a patient population that is consistent with these trial cohorts, in order to allow comparison of outcomes of SFRT with those in prior trials. Extraskeletal osteosarcoma should be excluded because of its rarity.

Grade 2-3 histologies are eligible. Grade 1 sarcoma and other histologies, including rhabdomyosarcoma, Ewing's sarcoma, chondrosarcoma, Kaposi's sarcoma and angiosarcoma should be excluded (high consensus). While some of these histologies have been treated with SFRT, their different natural disease course and rarity was deemed to add confounding variability to a clinical trial of SFRT cohort.

Inclusion of more aggressive sarcomas histologies should be considered in a future study, as defined in RTOG 9514 ^16^ to build upon the prior clinical work. The redundancy in molecular pathways makes molecular targeting in sarcomas difficult. Post-hoc stratification may be done based on molecular studies such as p53, Rb, EWS and non-EWS fusion proteins ^18^. However, for an initial study, practical consideration of recruitment (in view of the rarity of sarcoma), would make sub-classification or stratification based on molecular markers challenging.

*Eligibility/Exclusion criteria: Prior treatment*

Recurrent sarcomas after either previous resection or previous radiation therapy should be excluded to prevent confounding variables within a clinical trial.

*Eligibility/Exclusion criteria: Patient factors (age, toxicity risk factors)*

Patients with scleroderma who may have a high toxicity risk from radiation, particularly in subcutaneous and skin regions, should be excluded from a clinical trial (high consensus). Patient age should follow general trial criteria, and an upper age limitation of 85 years may be appropriate (moderate consensus).

*Stratifications*

Patients should be stratified by tumor bulk, using largest imaging-based tumor diameter, of <12 cm vs. >12 cm. If neoadjuvant chemotherapy is used (see section *Concurrent systemic therapy: Agents and timing*), then neoadjuvant chemotherapy vs. no chemotherapy should be stratified, as both regimens are in clinical use.

*Endpoints*

The feasibility of delivering SFRT according to the dosimetric and physics specifications ^19^ (see section *Radiation Therapy: SFRT dose*), and response metrics including primary tumor response, classified by imaging and by pathology response criteria, and resectability are suitable potential primary endpoints. Local recurrence-free, metastasis-free and overall survival, and quality of life outcomes present additional clinical trial endpoints.

*Pre-treatment Evaluations (clinical, imaging, histologic investigations)*

Standard workup with, preferably with MRI and or CT of the involved site is recommended. For metastatic workup chest abdomen and pelvis CT and PET/CT were recommended.

*Radiation Therapy – SFRT: Dose*

A dose range of 15 to 18 Gy in 1 fraction was considered appropriate as the dose regimen for clinical trials (high consensus), with the higher dose favored. Prescription parameters for deep-seated tumors and dosimetric characteristics to capture the heterogeneous dose distribution are further described in the recent GRID Physics and Dosimetry white paper ^19^. The EUD of the SFRT regimen must be reported for tumor and normal tissues using EUD calculation methods provided in the literature ^19^ . A modified linear quadratic (MLQ) based EUD calculation is favored because of its greater accuracy at doses of >10 Gy for non-uniform fields.

*Radiation Therapy – SFRT: Target volume*

The target volume for SFRT, based on clinical experience ^4-6^, is the GTV of the primary tumor, without an additional margin.

*Radiation Therapy – SFRT: OAR constraints*

Consideration should be given to exclude sensitive neural structures such as brachial plexus from the SFRT target volume and the beam path (high consensus). Exclusion of OARs with a 1 cm margin through collimation and MLC blocking are expected to achieve negligible SFRT dose to the recommended dose limit of <0.5Gy. It is also recognized that this may not be possible if these OARs are involved with tumor.

The skin surface dose from the SFRT should be <150% of the prescribed GRID dose or a maximum of 30 Gy (150% of 20 Gy) based on prior brachytherapy experience in STS ^20^. Because of the skin’s tolerance of high dose to a small volume, tolerance of the GRID dose is generally expected. However, in some bulky sarcomas the skin may be already stretched at the time of presentation, compromising vascularity.

*Radiation Therapy – SFRT: Technique*

For an initial clinical trial, it is recommended that GRID therapy be the technology of choice, based on available clinical experience with GRID therapy ^1 2 4-6^ and the currently insufficient clinical experience with Lattice therapy in sarcoma (high consensus). Lattice therapy may be appropriate in future trials.

*Radiation Therapy – Conventional ERT: Dose and technique*

There is high consensus that the conventional ERT dose, following the SFRT fraction, should be 50 Gy in 25-28 fractions to the PTV, per RTOG trial regimens using IMRT or 3D Conformal technique ^12 17^. As in standard-of-care radiotherapy, treatment to the entire extremity circumference is to be avoided (high consensus). Most commonly the conventional ERT course begins 1-2 days after SFRT ^3^ and should start ideally within 3 days of the SFRT fraction. The interval between the SFRT and conventional ERT should be documented to elucidate if treatment outcome may be affected by the length of interval.

*Radiation Therapy – Conventional ERT: OAR constraints*

Conventional dose constraints to critical normal tissues should be applied. The dose contribution from the SFRT should not be counted towards the dose constraints (moderate consensus). If there is concern regarding normal tissue doses, the dose to normal structures should be reduced *upfront* by adjusting field size and shape (usually through a secondary collimator such as MLC), thus changing the dose coverage in the SFRT fraction.

Dose contributions from SFRT and conventional ERT are reported separately because of the difficulty of summating the highly heterogeneous SFRT dose and the more uniform conventional dosing into a single dosimetric parameter. The EUDs from the SFRT to the target and the normal structures (usually outside of the target volume and estimated based on the SFRT valley dose and distance to the edge of SFRT field) should be added to that of the conventional ERT. These dose metrics and corresponding EUD can be employed for clinical correlation with tumor control and toxicity.

*On-therapy Evaluations and feasibility*

On-treatment evaluations should consist in standard weekly toxicity assessments, quality of life assessments and patient reported outcomes. Specimen collection of blood and urine multiple times during radiation therapy for the design of translational correlative of studies to investigate the underlying mechanisms should be considered. While serial blood draws during the treatment course are not standard-of-care in radiation therapy for sarcoma, collection of blood and urine for correlative studies is considered acceptable and feasible for a clinical trial (high consensus). Tumor biopsies during the treatment course for the purpose of correlative studies was considered to be not clinically feasible (high consensus).

*Systemic Therapy: Agents and timing*

Any neoadjuvant prior to the radiation therapy course and adjuvant chemotherapy following radiation therapy completion is acceptable, reflecting an acceptance current varying practice patterns in bulky soft tissue sarcoma. Agents considered acceptable in standard-of-care practice are allowed in clinical trials (high consensus).

Concurrent chemotherapy, delivered *during* the radiation therapy course, is not permitted in an initial clinical trial (high consensus). Concurrent or interdigitated chemotherapy is inconsistently and not widely used in current clinical practice, providing a rationale for its omission, as concurrent chemotherapy may also introduce confounding variables in the interpretation of response, toxicity and overall outcome results.

*Systemic Therapy: Immunotherapy*

Immunotherapy is not recommended in an initial clinical trial in order to reduce variables that may confound endpoints in an initial clinical trial (high consensus). However, immunotherapy can be studied in subsequent trials or as part of a lead-in study as more combined radiotherapy and immunotherapy data emerge in sarcoma.

*Post-radiation Therapy (preoperative) Evaluations:  Response assessment*

For post-radiation therapy, preoperative response assessment, preferably MRI-based assessment of imaging response per RECIST criteria is recommended and quantitative assessment of tumor necrosis should be documented (high consensus). Standard clinical examination (high consensus) should also be noted. A time interval of 4-8 weeks post-radiation is recommended for post-radiation/preoperative response assessment. These assessments should be done in conjunction with patient reported outcome assessments that include quality of life (high consensus).

*Surgical Evaluation, Pathologic response*

Pathologic tumor response, as carried out routinely in standard of care, provides as an important outcome assessment for SFRT response in clinical trials for soft tissue sarcoma. The surgical specimen further provides an important potential resource for the prospective study of

post-SFRT molecular markers in both the irradiated tumor and normal tissue. Special attention should be paid to tumoral specimen orientation for potential identification of tissue exposed to GRID peaks and valleys.

In the post-radiation-therapy assessment, criteria that should be collected are negative-margin resectability, R0 and R1 resection, and by pathologic criteria of tumor response, including quantitative histologic assessment of necrosis of >90% (high consensus).

*Post-therapy Evaluations (after completion of all therapy)*

Consensus on response and toxicity evaluations after completion of all therapies was high, and follows the general standard of care. Follow-up evaluations should occur every 3-4 months for the first 2 years post-therapy; every 6 months for 3 years, and subsequently yearly. These evaluations consist of clinical examination combined with imaging, generally MRI, specific to the primary site of the sarcoma, and CT imaging as clinically indicated. History and clinical exam are indispensable for the assessment of function and toxicity. Patient reported outcomes, including quality of life assessments should be combined with the routine post-therapy evaluations.

**Knowledge Gaps that May be Addressed through SFRT Clinical Trials in sarcoma**

*Clinical* knowledge gaps identified by consensus voters and Expert Panel include a better understanding of the effectiveness of SFRT in increasing pathologic CR rates; potential local and systemic effects; and local long term toxicity of SFRT in sarcoma.

Knowledge gaps in the *physics* of SFRT focus on further understanding of appropriate field set up, treatment delivery and quality assurance.

Knowledge gaps in area of *biology* include the wide range of biologic effect of SFRT.

**Conclusion**

SFRT clinical trials in Sarcoma are feasible based on the clinical experience provided by the pilot studies. Recommendations for eligibility aim to establish a uniform patient cohort of bulky extremity sarcomas who are planned to be treated with preoperative radiation with or without neoadjuvant chemotherapy. Less common trunk and other primary site locations and less common histologies should be excluded to support adequate patient enrollment while minimizing confounding variables that may hamper the interpretation of the outcome results. GRID technology is favored over Lattice radiotherapy based the technologies used in the current pilot studies. A single SFRT fraction of 15-18 Gy is recommended, and is followed by full-dose conventional (uniform) preoperative external beam radiation therapy. Reporting of inhomogeneity dose parameters according to recent SFRT physics guidelines, particularly EUD is highly recommended to allow data interpretation, plan comparison and correlation of dose parameters with clinical outcome. Chemotherapy agents that are used in standard-of-care management are permitted for the use of neoadjuvant or adjuvant chemotherapy. Concurrent chemotherapy is not permitted. Pre-therapy, on-therapy and post-therapy investigations to assess tumor control and toxicity endpoints generally follow the standard of care and should include patient reported outcomes. Specimen collection (blood, urine), synchronized prospectively with the treatment course, for translational correlative science studies is highly recommended. Systematic post-radiation pre-operative imaging assessment and pathologic tumor response assessment from definitive resection follow the standard criteria and include quantitative assessment of tumor necrosis. While pre-therapy (diagnostic) biopsies and tissue procurement from the post-radiotherapy/surgical resection specimens provide a potential resource for the prospective study of correlative molecular tissue markers, tumor tissue collection during the radiation therapy course for correlative science is challenging.

**References:**

1. Mohiuddin M, Fujita M, Regine WF, et al. High-dose spatially-fractionated radiation (GRID): a new paradigm in the management of advanced cancers. *Int J Radiat Oncol Biol Phys* 1999;45(3):721-7. doi: 10.1016/s0360-3016(99)00170-4

2. Mohiuddin M, Stevens JH, Reiff JE, et al. Spatially fractionated (GRID) radiation for palliative treatment of advanced cancer. *Radiation Oncocogy Investigations* 1996;4:41-47.

3. Neuner G, Mohiuddin MM, Vander Walde N, et al. High-dose spatially fractionated GRID radiation therapy (SFGRT): a comparison of treatment outcomes with Cerrobend vs. MLC SFGRT. *Int J Radiat Oncol Biol Phys* 2012;82(5):1642-9. doi: 10.1016/j.ijrobp.2011.01.065

4. Mohiuddin M, Memon M, Nobah A, et al. Locally advanced high-grade extremity soft tissue sarcoma: Response with novel approach to neoadjuvant chemoradiation using induction spatially fractionated GRID radiotherapy (SFGRT) (abstr). J Clin Oncol 2014;32:10575. *J Clin Oncol* 2014;32:10575.

5. Mohiuddin M, Miller T, Ronjon P, et al. Spatially fractionated grid radiation (SFGRT): A novel approach in the management of recurrent and unresectable soft tissue sarcoma (abstr). *Int J Radiat Oncol Biol Phys* 2009;75:S526.

6. Snider JW, Molitoris J, Shyu S, et al. Spatially Fractionated Radiotherapy (GRID) Prior to Standard Neoadjuvant Conventionally Fractionated Radiotherapy for Bulky, High-Risk Soft Tissue and Osteosarcomas: Feasibility, Safety, and Promising Pathologic Response Rates. *Radiat Res* 2020 doi: 10.1667/rade-20-00100.1 [published Online First: 2020/10/17]

7. Huhn JL, Regine WF, Valentino JP, et al. Spatially fractionated GRID radiation treatment of advanced neck disease associated with head and neck cancer. *Technol Cancer Res Treat* 2006;5(6):607-12. doi: 10.1177/153303460600500608

8. Penagaricano JA, Moros EG, Ratanatharathorn V, et al. Evaluation of spatially fractionated radiotherapy (GRID) and definitive chemoradiotherapy with curative intent for locally advanced squamous cell carcinoma of the head and neck: initial response rates and toxicity. *Int J Radiat Oncol Biol Phys* 2010;76(5):1369-75. doi: 10.1016/j.ijrobp.2009.03.030

9. Choi JI, Daniels J, Cohen D, et al. Clinical Outcomes of Spatially Fractionated GRID Radiotherapy in the Treatment of Bulky Tumors of the Head and Neck. *Cureus* 2019;11(5):e4637. doi: 10.7759/cureus.4637

10. Eilber FC, Rosen G, Eckardt J, et al. Treatment-induced pathologic necrosis: a predictor of local recurrence and survival in patients receiving neoadjuvant therapy for high-grade extremity soft tissue sarcomas. *J Clin Oncol* 2001;19(13):3203-9. doi: 10.1200/JCO.2001.19.13.3203

11. DeLaney TF, Spiro IJ, Suit HD, et al. Neoadjuvant chemotherapy and radiotherapy for large extremity soft-tissue sarcomas. *Int J Radiat Oncol Biol Phys* 2003;56(4):1117-27. doi: 10.1016/s0360-3016(03)00186-x [published Online First: 2003/06/28]

12. Wang D, Zhang Q, Eisenberg BL, et al. Significant Reduction of Late Toxicities in Patients With Extremity Sarcoma Treated With Image-Guided Radiation Therapy to a Reduced Target Volume: Results of Radiation Therapy Oncology Group RTOG-0630 Trial. *J Clin Oncol* 2015;33(20):2231-8. doi: 10.1200/jco.2014.58.5828 [published Online First: 2015/02/11]

13. Kepka L, DeLaney TF, Suit HD, et al. Results of radiation therapy for unresected soft-tissue sarcomas. *Int J Radiat Oncol Biol Phys* 2005;63(3):852-9. doi: 10.1016/j.ijrobp.2005.03.004

14. Roberge D, Skamene T, Nahal A, et al. Radiological and pathological response following pre-operative radiotherapy for soft-tissue sarcoma. *Radiother Oncol* 2010;97(3):404-7. doi: 10.1016/j.radonc.2010.10.007

15. Wang D, Harris J, Kraybill WG, et al. Pathologic complete response and survival outcomes in patients with localized soft tissue sarcoma treated with neoadjuvant chemoradiotherapy or radiotherapy: Long-term update of NRG Oncology RTOG 9514 and 0630. *Journal of Clinical Oncology* 2017;35(15_suppl):11012-12. doi: 10.1200/JCO.2017.35.15_suppl.11012

16. Kraybill WG, Harris J, Spiro IJ, et al. Phase II study of neoadjuvant chemotherapy and radiation therapy in the management of high-risk, high-grade, soft tissue sarcomas of the extremities and body wall: Radiation Therapy Oncology Group Trial 9514. *J Clin Oncol* 2006;24(4):619-25. doi: 10.1200/JCO.2005.02.5577

17. Wang D, Bosch W, Roberge D, et al. RTOG sarcoma radiation oncologists reach consensus on gross tumor volume and clinical target volume on computed tomographic images for preoperative radiotherapy of primary soft tissue sarcoma of extremity in Radiation Therapy Oncology Group studies. *Int J Radiat Oncol Biol Phys* 2011;81(4):e525-8. doi: 10.1016/j.ijrobp.2011.04.038

18. Jain S, Xu R, Prieto VG, et al. Molecular classification of soft tissue sarcomas and its clinical applications. *Int J Clin Exp Pathol* 2010;3(4):416-28.

19. Zhang H, Wu X, Zhang X, et al. Photon GRID Radiation Therapy: A Physics and Dosimetry White Paper from the Radiosurgery Society (RSS) GRID-Lattice-Microbeam-FLASH Radiotherapy Working Group. *Radiat Res* 2020 doi: 10.1667/RADE-20-00047.1

20. Emory CL, Montgomery CO, Potter BK, et al. Early complications of high-dose-rate brachytherapy in soft tissue sarcoma: a comparison with traditional external-beam radiotherapy. *Clin Orthop Relat Res* 2012;470(3):751-8. doi: 10.1007/s11999-011-2106-5
